# Metabolite profiling and transcriptomic analyses reveal an essential role of UVR8-mediated signal transduction pathway in regulating flavonoid biosynthesis in tea plants (*Camellia sinensis*) in response to shading

**DOI:** 10.1186/s12870-018-1440-0

**Published:** 2018-10-12

**Authors:** Linlin Liu, Yingying Li, Guangbiao She, Xianchen Zhang, Brian Jordan, Qi Chen, Jian Zhao, Xiaochun Wan

**Affiliations:** 1 State Key Laboratory of Tea Plant Biology and Utilization, Anhui Agricultural University, Hefei, 230036 Anhui China; 2 Centre for Viticulture and Oenology, Faculty of Agriculture and Life Sciences, Lincoln University, Christchurch, 7647 New Zealand

**Keywords:** *Camellia sinensis*, Flavonoids, UVR8, UV-B responses, Shading, Light signal transduction

## Abstract

**Background:**

Tea is the most popular nonalcoholic beverage worldwide for its pleasant characteristics and healthful properties. Catechins, theanine and caffeine are the major natural products in tea buds and leaves that determine tea qualities such as infusion colors, tastes and fragrances, as well as their health benefits. Shading is a traditional and effective practice to modify natural product accumulation and to enhance the tea quality in tea plantation. However, the mechanism underlying the shading effects is not fully understood. This study aims to explore the regulation of flavonoid biosynthesis in *Camellia sinensis* under shading by using both metabolomic and transcriptional analyses.

**Results:**

While shading enhanced chlorophyll accumulation, major catechins, including C, EC, GC and EGC, decreased significantly in tea buds throughout the whole shading period. The reduction of catechins and flavonols were consistent with the simultaneous down-regulation of biosynthetic genes and TFs associated with flavonoid biosynthesis. Of 16 genes involved in the flavonoid biosynthetic pathway, *F3’H* and *FLS* significantly decreased throughout shading while the others (*PAL*, *CHSs*, *DFR*, *ANS, ANR* and *LAR*, etc.) temporally decreased in early or late shading stages. Gene co-expression cluster analysis suggested that a number of photoreceptors and potential genes involved in UV-B signal transductions (*UVR8_L*, *HY5*, *COP1* and *RUP1/2*) showed decreasing expression patterns consistent with structural genes (*F3’H*, *FLS*, *ANS*, *ANR*, *LAR*, *DFR* and *CHSs*) and potential TFs (*MYB4, MYB12*, *MYB14* and *MYB111*) involved in flavonoid biosynthesis, when compared with genes in the UV-A/blue and red/far-red light signal transductions. The KEGG enrichment and matrix correlation analyses also attributed the regulation of catechin biosynthesis to the UVR8-mediated signal transduction pathway. Further UV-B treatment in the controlled environment confirmed UV-B induction on flavonols and EGCG accumulation in tea leaves.

**Conclusions:**

We proposed that catechin biosynthesis in *C. sinensis* leaves is predominantly regulated by UV through the UVR8-mediated signal transduction pathway to MYB12/MYB4 downstream effectors, to modulate flavonoid accumulation. Our study provides new insights into our understanding of regulatory mechanisms for shading-enhanced tea quality.

**Electronic supplementary material:**

The online version of this article (10.1186/s12870-018-1440-0) contains supplementary material, which is available to authorized users.

## Background

Tea, one of the products processed from the leaves of tea plants (*Camellia sinensis* (L.) O. Kuntze), is the oldest and most popular nonalcoholic caffeine-containing beverage in the world [1]. Besides the pleasant characteristics, tea is famous for its numerous healthful and medicinal benefits due to many of the characteristic secondary metabolites in tea leaves [2, 3]. The medicinal benefits of tea are primarily attributed to catechins, including catechin (C), epicatechin (EC), gallocatechin (GC), epigallocatechin (EGC), and their respective gallate esters, epigallocatechin gallate (EGCG), epicatechin gallate (ECG) and gallocatechin gallate (GCG) [2, 3]. Numerous studies have indicated that catechins improve human health through their bioactive functions, such as anti-oxidant, anti-radiation and anti-bacterial properties [4, 5]. In addition to the medicinal properties, these compounds contribute to the color, aroma and mouth-feel of tea infusion, eventually determine the sensation characteristics of tea products [6, 7]. Particularly for black tea, galloylated catechins were shown to be the major contributors to the astringent and bitter sensation [6, 7], while flavonol 3-*O*-glycosides have been found to confer velvety and mouth-drying tastes [6]. However, the ratio of catechins to amino acids in tea buds and leaves is one of the critical parameters evaluating the tea quality [8]. Relatively low ratio of catechins to amino acids advances tea infusion in freshness and umami, favored by tea consumers [8, 9]. Therefore, to achieve a good balance between the accumulation of catechins and amino acids in consideration of both the tea sensation and health benefits is primarily important for tea industry, which is also a practical objective for tea research.

In tea plantation, shading is one of the traditional agricultural practices to improve tea characteristics through modifying the ratio of catechins and free amino acids in tea leaves [10–12]. Taking *C. sinensis* cv. *Shuchazao* as an example, the accumulation of catechins reduced under shading conditions along with the simultaneous down-regulation of biosynthetic genes involved in flavonoid biosynthesis [11, 13]. Compared to the sunlight-exposed leaves, the contents of catechins and flavonols in shaded leaves (20 ± 5% light transmitting) decreased by more than 50% and 40%, respectively [11]. Meanwhile, the transcript levels of genes encoding key enzymes involved in the flavonoid biosynthetic pathway, including phenylalanine ammonialyase (*PAL*), flavonoid 3′-hydroxylase (*F3’H*), dihydroflavonol reductase (*DFR*), anthocyanidin reductase (*ANR*), chalcone synthase (*CHS*) and flavonoid 3′, 5′-hydroxylase (*F3’5’H*) are notably reduced [11, 13]. In the majority of previous studies, changes of secondary metabolites under shading were attributed to a reduction of light intensity reached tea plants [11–14]. However, the mechanism underlying the shading effects on plant secondary metabolism and photomorphogenesis can be specific and sophisticated, in relation to responses by various photoreceptors and light signal transductions [15–18].

When emerging underneath an established canopy or shading condition, plants have to perceive transient and actual light quality and quantity through a variety of informational photoperceptions and light signals, including reduced red: far-red (R:FR) ratio and reduced irradiances of ultraviolet (UV) [17, 18]. This mechanism involves various photoreceptors and early signaling events in response to differential light signals [15, 19], including phytochromes (PHYs, PHYA-PHYE) for the red/far-red light signal transduction [20–22], and cryptochromes (CRYs, CRY1 and CRY2), phototropins (PHOTs, PHOT1 and PHOT2) and zeitlupes family (ZTL) for the blue/UV-A light signal transduction [23, 24], whereas UV RESISTANCE LOCUS8 (UVR8) for the low fluence UV-B perception and signal transductions [25–27]. These photoreceptors provide plants with information about the changes of growth conditions and modulate the expression of adaptive morphological and physiological responses [16, 21, 28]. Once under a canopy or shading condition, light transmitted through or reflected by plant leaves has a low ratio of R:FR and reduced UV-B radiation [17, 18]. In response to these conditions, a variety of photoreceptors participate in the perception of actual light therefore lead to changes in plant morphogenesis and activation of the shade-avoidance syndrome [15, 18]. It has been suggested that UVR8-mediated responses are likely to be involved in plant morphological responses due to UV-B attenuation under shading conditions [15–17]. However, no attention has been paid to the UVR8-mediated signal transduction in *C. sinensis*, although a previous study showed that low fluence and short term of UV-B radiation increased EGCG accumulation in tea plants [29].

In the model plant *Arabidopsis thaliana*, photoreceptor-targeted bZIP transcription factor (TF) ELONGATED HYPOCOTYL 5 (HY5), a central modulator for light signal transductions and shoot-to-root signal transducer [17, 19], has been shown to play a crucial role in regulating flavonoid accumulation in response to UV-B radiation [30, 31]. It is reported that HY5 regulates the expression of MYB12, a specific TF for flavonol synthase (*FLS*), to determine flavonoid accumulation in response to light and UV-B radiation [30]. Another UV-inducible TF MYB4 acts as a transcriptional repressor of cinnamate 4-hydroxylase (*C4H*), 4-coumarate-CoA ligase (*4CL*), leucoanthocyanidin reductase (*LAR*), *CHS* and *ANR2*, to mediate UV-B-dependent phenylpropanoid and anthocyanin biosynthesis in *A. thaliana*, *Brassica rapa* and *C. sinensis* [32–34]. Furthermore, evidences showed three flavonol-specific regulators MYB11, MYB12 and MYB111 activate, in parallel, the biosynthetic enzyme encoding genes *CHS*, *FLS*, *F3’H* and chalcone isomerase (*CHI*) for flavonol accumulation and tissue distribution in *A. thaliana* [35, 36]. These MYB TFs are suggested to bind a similar light regulatory unit (consisting of a MYB-recognition element and an ACGT-containing element) in the promoters of different target genes even without a known bHLH partner for activation [36–38]. All data raise the possibility that HY5 activates in an early step expressions of MYB TFs to subsequently work in concert with these TFs on delivering light information from UVR8-mediated early signal events to flavonoid biosynthesis [30, 38].

To date, there has been no study that considers light signal transductions at the molecular level in the commercially significant plant, *C. sinensis*. This research aims to investigate the roles of different light signals in determining flavonoid biosynthesis in response to shading. In light of the importance of flavonoids to tea characteristics, we investigated the flavonoid accumulation in tea buds in response to shading, the temporal regulation of gene and TF activities involved in flavonoid biosynthesis, and potential light signal transduction pathways including the UVR8-mediated signal transduction pathway, UV-A/blue light and red/far-red light signal transduction pathways, as important signal regulators of gene activities in tea plants. Based on the data from both the shading treatments in tea plantation and UV-B experiment in the controlled environment, we propose a regulatory mechanism in which UVR8-mediated UV-B detection under shading condition down regulates HY5 accumulation in tea buds, which then inhibits the expression of MYB12 to reduce downstream responsive gene activities in the flavonoid biosynthetic pathway, therefore limits flavonoid accumulation in tea plants. These results improve our understanding of secondary mechanism regulatory in tea plants and also provide new insights into the participation of different light signals in determining secondary metabolites in important commercial species.

## Results

### Flavonoid changes in tea buds under shading conditions

**Fig. 1:**
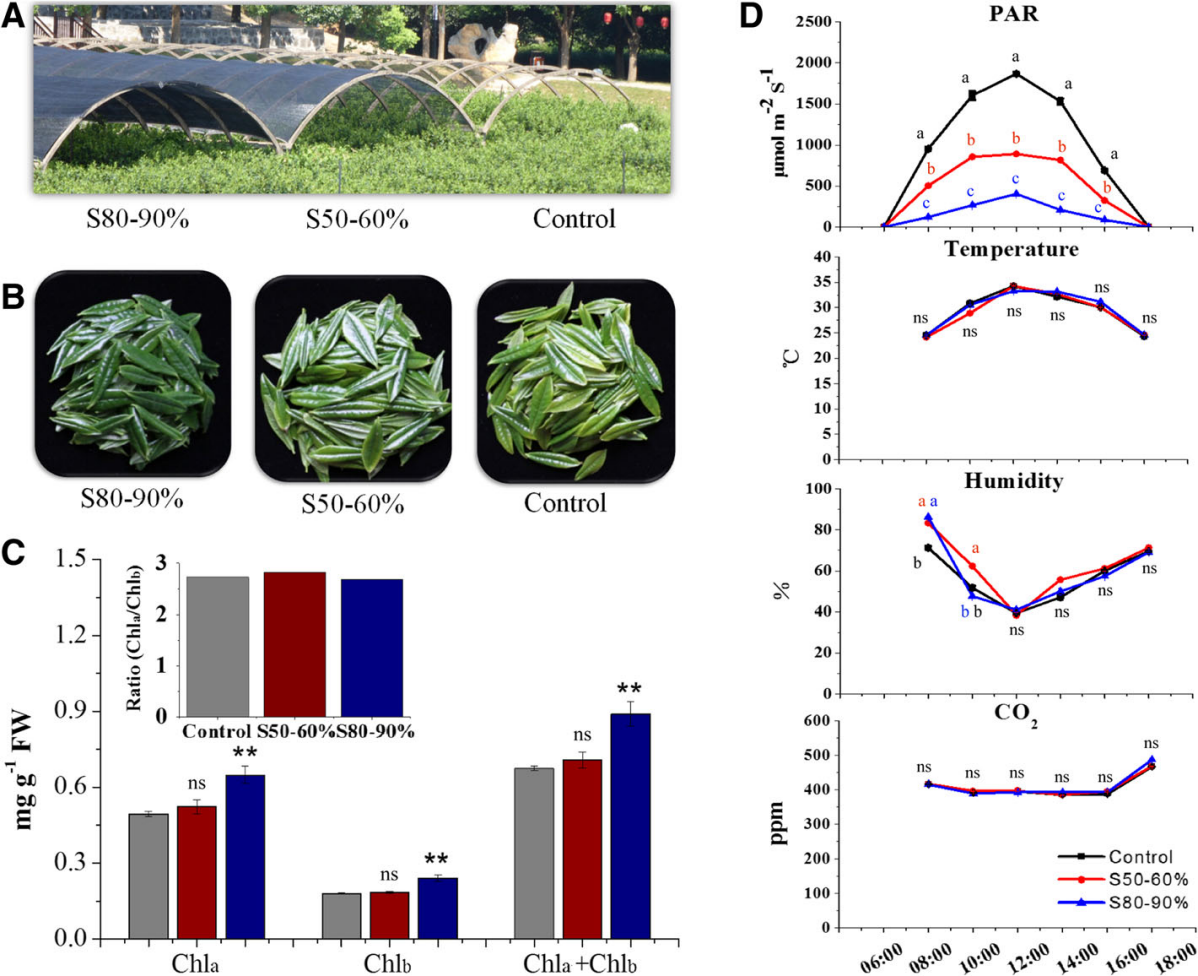
The effects of shading on chlorophylls and daily environmental parameters in shading experiment in tea plantation. **a** The set up of shading treatments in tea plantation. **b** The effects of shading on appearance of tea leaves after 14 days. **c** The effects of shading on chlorophylls after 14 days. **d** The effects of shading on daily environmental parameters among different treatments in tea plantation. The treatments are: tea plants with naturally growth (Control); tea plants with 50–60% shading treatment (S50–60%); tea plants with 80–90% shading treatment (S80–90%). PAR, photosynthetic actively radiation; Chl_a_, chlorophyll a; Chl_b_, chlorophyll b; ns, no significance. Data shown are the average mean ± SE of three replicates (*n* = 3). *Significant differences comparing the Control treatment at each time point according to one-way analysis of variance (ANOVA) test and a Fisher’s least significant difference (LSD) at the 5% significance level (*p < 0.05, **p < 0.01). Different letters (a, b, c) indicate statistical significance among treatments using one-way ANOVA and a Fisher’s LSD test at the 5% significance level

To investigate the effects of shading on tea plants, both medium and heavy shading treatments (S50–60% and S80–90%) were carried out on tea plants in plantation (Fig. 1a). Significant changes were observed in the appearance of tea leaves between different treatments. Tea leaves under shading conditions presented a darker green color and showed significant increases in chlorophylls, when compared with leaves in the control treatments (Fig. 1b and c). The contents of chlorophyll a and chlorophyll b increased but no significant change was detected in the ratio of chlorophyll a: chlorophyll b. The daily environmental factors were measured to monitor growth conditions of tea plants among different treatments in plantation (Fig. 1d). Excepting the photosynthetically active radiation (PAR), shading caused no significant change on other environmental factors, including the temperature, humidity and CO_2_ content.

**Fig. 2:**
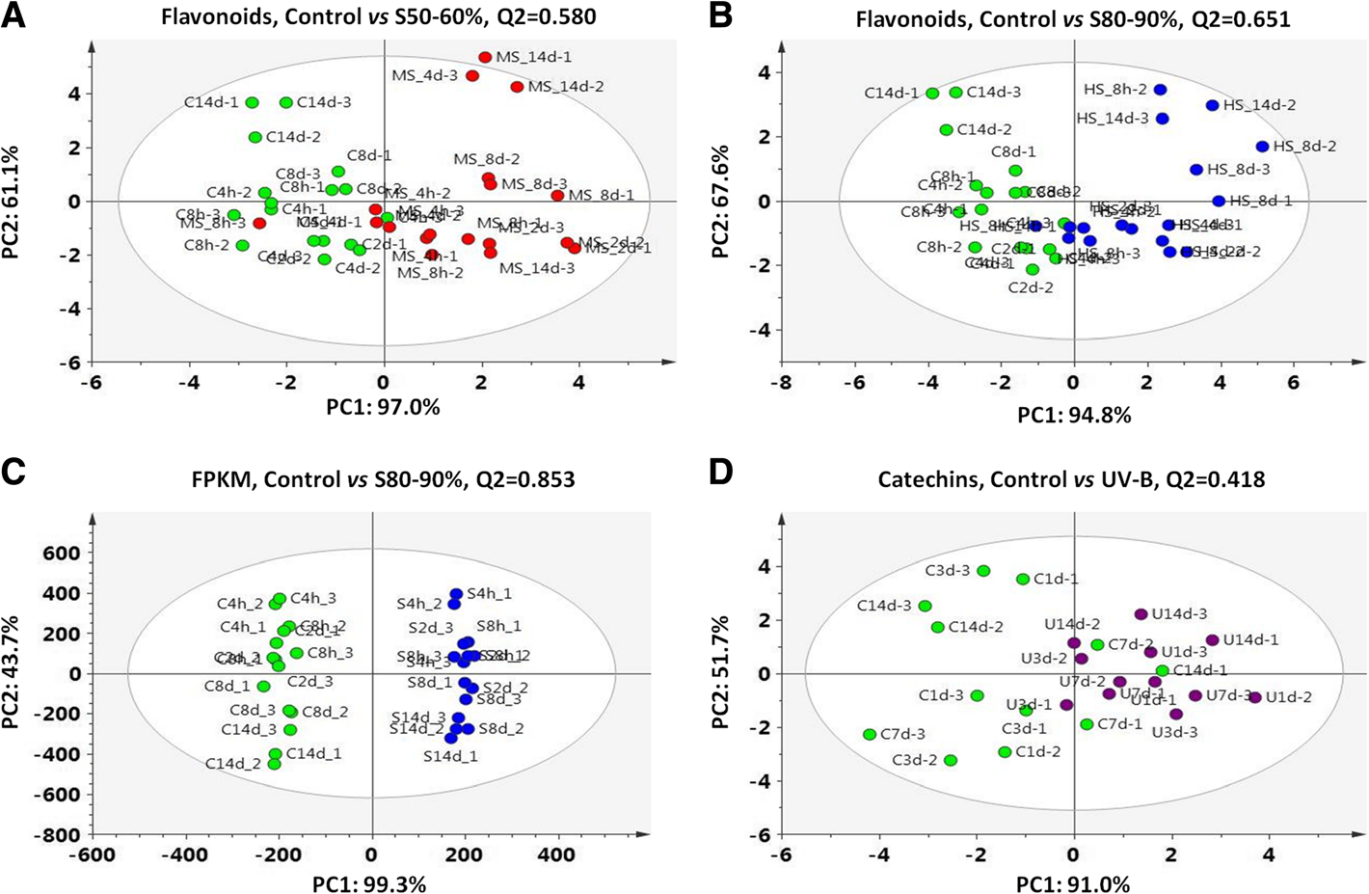
The OPLS-DA analysis of flavonoids and gene expression in tea buds from shading and UV-B experiments. **a** The OPLS-DA analysis of flavonoids in tea buds at different time points throughout shading period between the Control and medium shading (S50–60%) treatments. **b** The OPLS-DA analysis of flavonoids in tea buds at different time points throughout shading period between the Control and heavy shading (S80–90%) treatments. **c** The OPLS-DA analysis of transcript abundance of all unigenes annotated in transcriptome datasets between the Control and S80–90% treatments. **d** The OPLS-DA analysis of major catechins in tea buds between the Control and UV-B treatments in the controlled environment. Treatments in the shading experiment in tea plantation are shown as above in Fig. 1. Treatments in the UV-B experiment in the controlled environment are: tea plants exposed to pure PAR (Control); tea plants exposed to PAR + UV-B radiation (UV-B). FPKM, Fragment Per Kilo base of exon model per Million mapped reads. Data shown are from the value of three replicates (*n* = 3). OPLS-DA analysis was conducted by SIMCA 13.0 (UMETRICS, https://umetrics.com/)

**Fig. 3:**
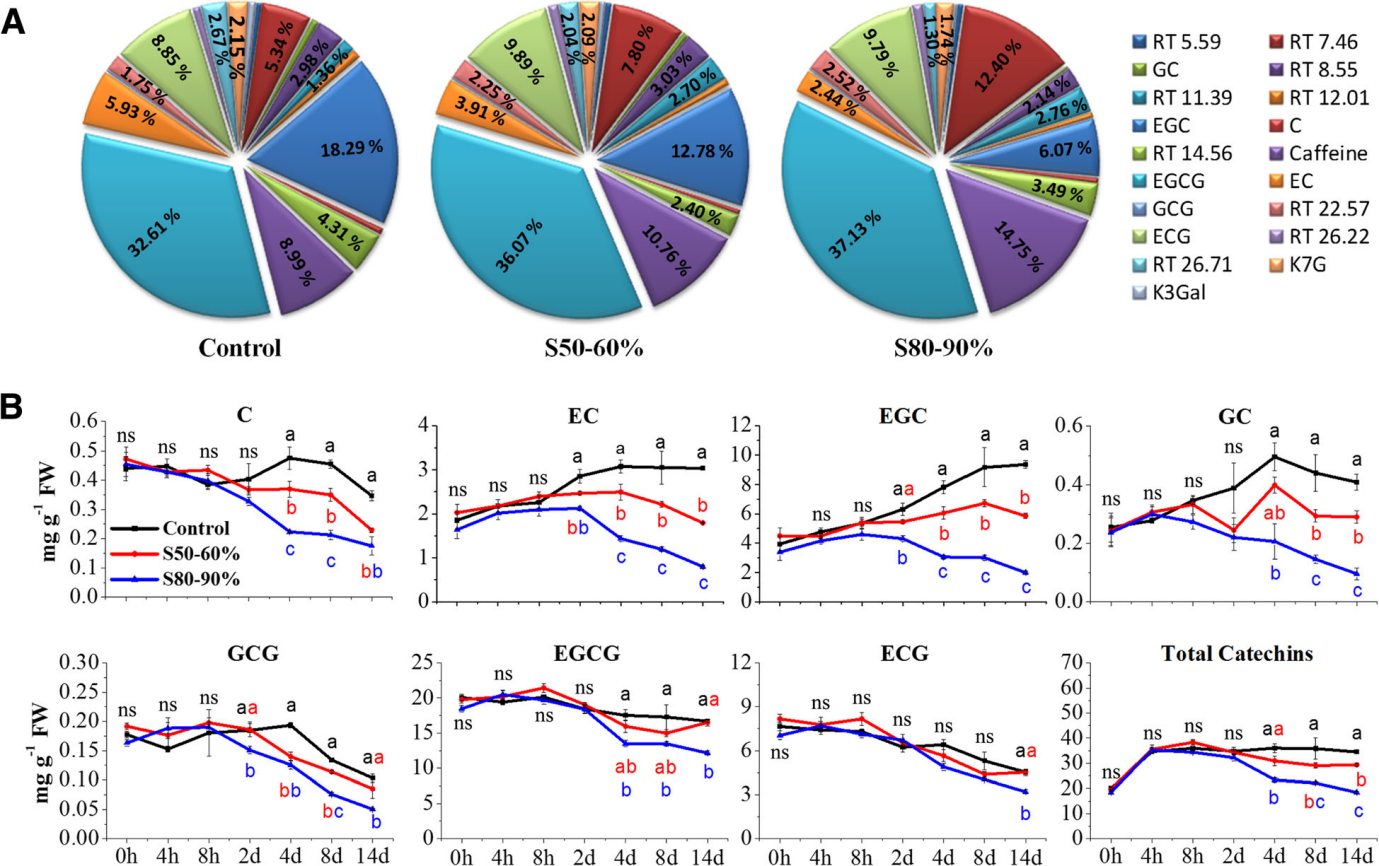
The effects of shading on flavonoid accumulation in tea buds in tea plantation. **a** The composition of flavonoids in tea buds from the control and shading treatments at time point of 14d. **b** The changes of major catechins in tea buds in different treatments throughout shading period. C, catechin; EC, epicatechin; EGC, epigallocatechin; GC, gallocatechin; GCG, gallocatechin 3-*O*-gallate; EGCG, epigallocatechin 3-*O*-gallate; ECG, epicatechin 3-*O*-gallate; K7G, keampferol-7-*O*-glucoside; K3Gal, kaempferol-3-*O*-galactopyranoside; RT, retention time; ns, no significance. Data shown are the average mean ± SE of three replicates (***n*** = 3). Different letters (a, b, c) indicate statistical significance among different treatments according to one-way ANOVA and a Fisher’s LSD test at the 5% level

To examine the shading effect on flavonoid accumulation, tea buds were sampled at different time points (from 4h to 14d) throughout shading treatments and were analysed with high-performance liquid chromatographic (HPLC, Figs. 2 and 3). Metabolite profiling showed that both shading treatments (S50–60% and S80–90%) significantly affected flavonoid accumulation in tea buds (Fig. 2a and b). Two flavonols, identified as keampferol-7-*O*-glucoside (K7G) and kaempferol-3-*O*-galactopyranoside (K3Gal), showed a reduction in proportion under shading condition when compared with controls. As the major flavonoids, catechin contents significantly reduced in response to shading treatments. The each component of major catechins, including C, EC, GC, EGC, ECG, EGCG and GCG, displayed distinct accumulation patterns over the shading treatments. Taking tea buds after 14 days of shading as an example, the significant changes in flavonoid composition were observed (Fig. 3a). After 14 days of shading, the proportion of GC, EGC, C and EC showed significant decreases in both S50–60% and S80–90% treatments, as compared with controls. In contrast, the catechin-3-*O*-gallates, including EGCG, ECG and GCG, showed increases to different extents.

In addition to the changes in flavonoid composition, significant changes were detected in both the total and individual catechins in tea buds throughout the whole shading period (Fig. 3b). As shown in Fig. 3b, the total catechins showed a significant decrease from 4h to 14d in both S50–60% and S80–90% treatments, and the decrease positively correlated to the levels and durations of shading. Similar pattern was observed in the accumulation of individual catechins, including C, EC, GC, and EGC. However, the catechin gallates presented no dramatic decrease. ECG showed no significant change in response to shading until at 14d, whereas GCG and EGCG only showed a minor decrease in the heavy shading treatment.

### KEGG enrichment of candidate pathways in response to shading

To investigate the effects of shading on metabolic biosynthesis of flavonoids at the transcriptional levels, we utilized RNA-Seq technology to analyse gene activities in tea buds from both the control and S80–90% treatments. After removing adaptor sequences, duplication sequences, ambiguous reads and low-quality reads, an average of 6 Gb clean reads per sample was generated. The final assembly of tea samples had 82322 unigenes (≥500 bp) with an N50 length of 1,206 bp. Functional annotation revealed 57823, 40003, 34066, 11963 and 34972 unigenes with alignments to the NR (Non-redundant protein database), Swiss-Prot (Annotated protein sequence database), KOG (Clusters of orthologous groups for eukaryotic complete genomes), KEGG (Kyoto encyclopedia of genes and genomes) and GO (Gene ontology) databases, respectively (Additional file 1). Statistic analysis of the transcript abundance of all unigenes annotated in this study showed a clear shading effect between the control and S80–90% treatment (Fig. 2c).

**Fig. 4:**
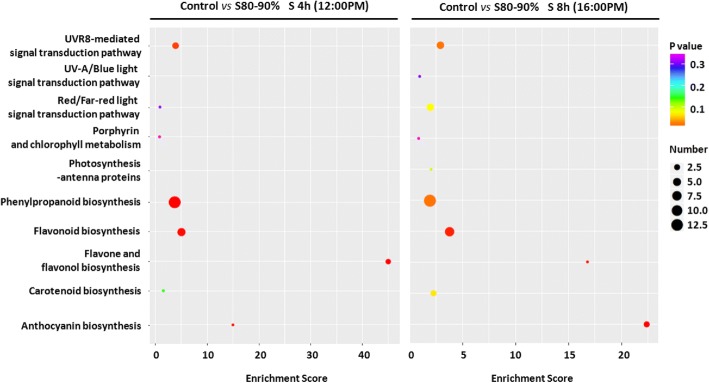
Functional distribution of the DEGs in candidate pathways in response to shading at time points of 4h and 8h. The enrichment score indicates intensiveness of DEGs (fold change ≥2) in a certain pathway (Enrichment score = (m/n): (M/N); m, the number of DEGs mapped to a certain pathway; n, the number of all DEGs annotated in transcriptome datasets; M, the number of unigenes mapped to a certain pathway; N, the number of all unigenes annotated in transcriptome datasets). A large enrichment score denotes a high degree of intensiveness. The *p* value (ranging from 0~1) was calculated using hypergeometric test through Bonferroni Correction and less *p* value means greater intensiveness. Gene number means number of DEGs mapped to a certain pathway according to KEGG database. S 4h and S 8h indicate time points at 4h and 8h throughout shading period

To study the roles of different light signal transduction pathways in regulating flavonoid accumulation in response to shading, we selected several candidate metabolic pathways involved in favonoid biosynthesis and light signal transductions to conduct the KEGG enrichment. In total 10 metabolic pathways with 825 potential unigenes involved in flavonoid biosynthesis, light signal transductions and photosynthesis were calculated for KEGG enrichment in this study (Fig. 4). Three light signal transduction pathways presented in this study were not registered in the KEGG database. Potential unigenes associated with these pathways, including specific photoreceptors and pigmentations of light perception, signal transduction mediators, transcription activators and repressors, as well as downstream genes and TFs involved in plants morphogenesis were collected from previous reports and complemented by the GO database. In addition to these light signal transduction pathways, specific emphasis was placed on related secondary metabolism involved in flavonoid biosynthesis, including the phenylpropanoid biosynthetic pathway (ko00940), flavonoid biosynthetic pathway (ko00941), and biosynthetic pathways for anthocyanin (ko00942), isoflavonoid (ko00943), and flavones and flavonol biosynthesis (ko00944). We also considered the flavonoid biosynthesis in the context of gene activities associated with the photosynthesis (Ko00195/ko00196) and chlorophyll metabolism (Ko00860/ko00906) in response to shading in tea plants.

As shown in Fig. 4, gene activities involved in the phenylpropanoid biosynthetic pathway showed more significant changes in response to shading when compared to other selected pathways, followed by the flavonoid biosynthetic pathway. Among three light signal transduction pathways, unigenes involved in the UVR8-mediated signal transduction pathway significantly changed in response to shading after 4h and 8h (samples were collected at 12:00 pm in noon for 4h and 16:00 pm in late afternoon for 8h, respectively). No significant change in gene transcripts involved in the UV-A/blue light signal transduction pathway was observed in response to shading at noon time and minor changes in the afternoon. Interestingly, unigenes involved in the red/far-red light signal transduction pathway showed slight shading effects in the middle of the day but more significant changes were observed in late afternoon. Genes involved in plant photosynthesis and chlorophyll metabolism showed less response to shading in this study, with similar findings among time points throughout shading period.

### Down-regulated gene expression associated with flavonoid biosynthesis in response to shading

**Fig. 5:**
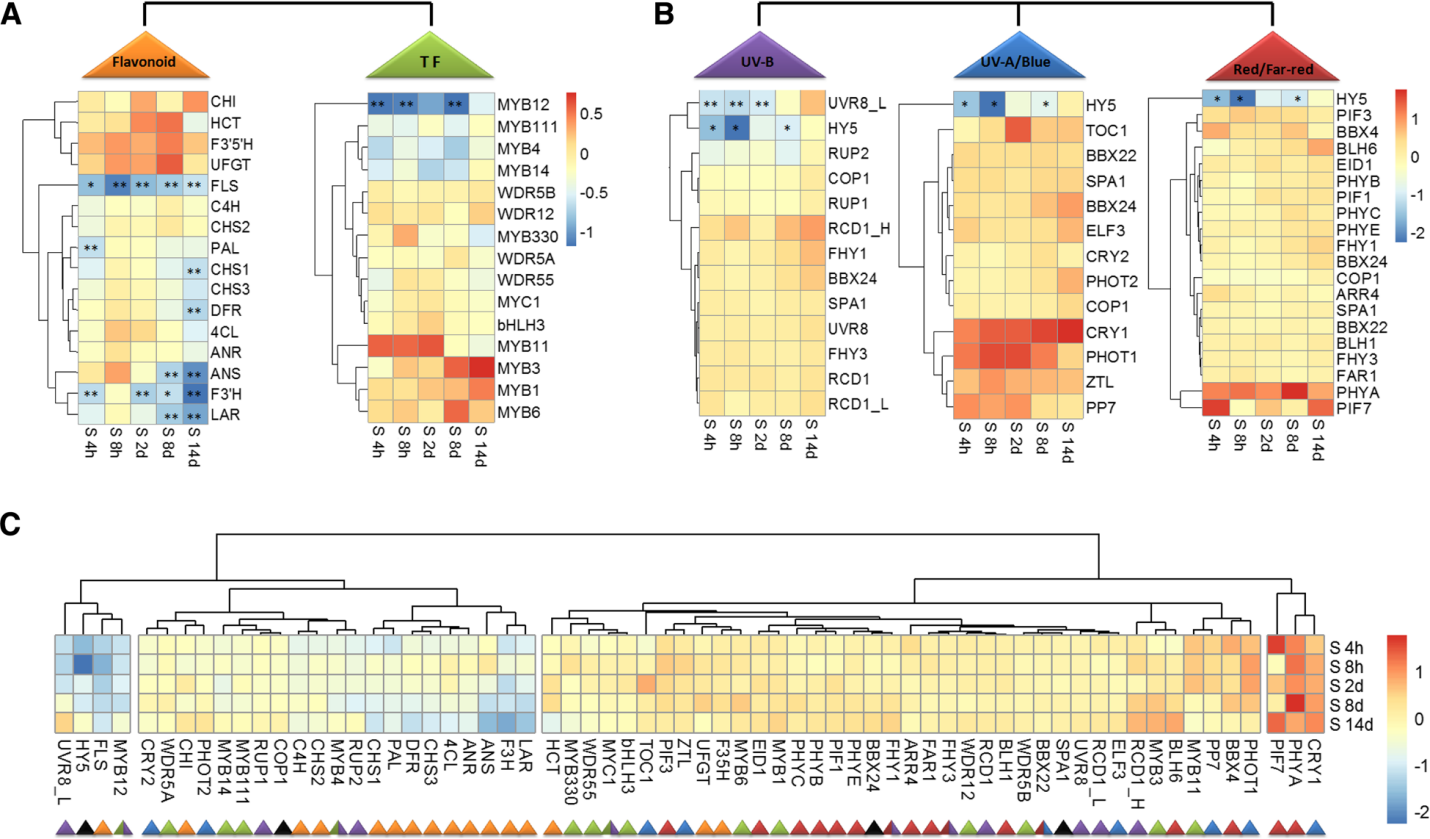
The shading effects on transcript abundance of potential genes and TFs involved in flavonoid biosynthesis and light signal transductions in tea buds. **a** The shading effects on transcript abundance of genes and potential TFs involved in the flavonoid biosynthetic pathway. **b** The shading effects on transcript abundance of potential genes and TFs involved in the UVR8-mediated UV-B, UV-A/blue light and red/far-red light signal transduction pathways, respectively. **c** Cluster analysis of expression of all potential genes and TFs in response to shading treatment. The heatmaps are constructed from the competitive expression of genes (log_2_ FPKM_S80–90%_/FPKM_control_) from the transcriptome datasets. S 4h, S 8h, S 2d, S 8d and S 14d indicate time points at 4h, 8h, 2d, 8d and 14d throughout shading period. The triangle with colors represents potential genes involved in the flavonoid biosynthetic pathway (functional enzymes, yellow; TFs, green) and different light transduction pathways (UV-B radiation, purple; UV-A/blue light, blue; red/far-red light, red; genes and TFs involved in three light signal transduction, black). Data shown are the average mean of three biological replicates (*n* = 3). *Significant differences comparing the Control treatment at each time point according to one-way ANOVA and a Fisher’s LSD test at the 5% level (*p < 0.05, **p < 0.01; fold change ≥2)

To explain the roles of different light signal transduction pathways in determining flavonoid accumulation in more details, the activities of unigenes and TFs involved in flavonoid biosynthesis and light signal transductions were analysed. We collected all annotated unigenes encoding each candidate gene and TF from our assembled tea transcriptome datasets (Additional files 2, 3, 4, 5 and 6), then selected one unigene as representative for each gene or TF and visualized the competitive expression (log_2_FPKM_S80–90%_/FPKM_control_; FPKM, Fragment Per Kilo base of exon model per Million mapped reads) throughout the shading treatment (Fig. 5). These representative unigenes were selected according to a comprehensive evaluation of parameters as following order: unigenes with best alignments to the reported sequence, the annotated unigenes with the highest FPKM value and unigenes differentially expressed (differentially expressed genes, DEGs), etc.

In total, 61 annotated unigenes encoding 16 major enzymes in the flavonoid biosynthetic pathway were annotated from our assembled tea transcriptome datasets (Additional file 2, Fig. 5a). From 4h to 14d throughout shading treatments, a group of unigenes catalyzing key enzymes in the flavonoid biosynthetic pathway showed significant decreases under shading condition. These unigenes were *F3’H* and *FLS*, which significantly decreased in transcript levels in tea buds throughout the whole period of shading. *PAL* showed a significant decrease immediately after 4 h of shading, but the transcript levels increased to normal levels in later shading period. In contrast, *CHS*, *DFR*, anthocyanidin synthase (*ANS*) and *LAR* unigenes, which directly produce catechins in late steps of the flavonoid biosynthetic pathway, presented no significant change during earlier shading period but significantly decreased after 8d and 14d of shading.

TFs potentially associated with the flavonoid biosynthetic pathway in tea plants were also examined to study the regulation of flavonoid biosynthesis in response to shading (Fig. 5a). According to the previous studies on the function of MYB-bHLH-WD40 (MBW) TF complex in regulating flavonoid biosynthesis in the model plant *A. thaliana* [30, 33–35], 26 annotated unigenes encoding 15 candidate TFs of MYB, bHLH and WD40 were annotated in our assembled tea transcriptome datasets (Additional file 3). These TFs included MYB1, MYB4, MYB6, MYB11, MYB12, MYB111 and MYB330, etc. However, most of these unigenes showed no significant change in response to shading treatments. *MYB12* is the only one showed significant decreases in tea buds throughout the whole shading treatment (Fig. 5a).

### Altered UV-B light signal transductions in response to shading

To explore the roles of different light signal transduction pathways in determining flavonoid biosynthesis in tea plants in response to shading, we investigated the activities of potential unigenes (including light photoreceptors, perception pigmentations, signal mediators and TFs) that thought to be involved in the UVR8-mediated low fluence UV-B responses, UV-A/blue light and red/far-red light signal transduction pathways in our assembled tea transcriptome datasets (Fig. 5b). These unigenes included 13 selected unigenes (in total 22 unigenes annotated in transcriptome datasets, Additional file 4) reported to be associated with the UVR8-mediated low fluence UV-B signal transduction pathway [25, 26, 39], 13 selected unigenes (23 unigenes annotated, Additional file 5) recognized to be involved in the UV-A/blue light signal transduction pathway [23, 24], and 20 selected unigenes (32 unigenes annotated, Additional file 6) thought to be related to the red/far-red light signal transduction pathway [22, 40]. The central players in light signaling pathways, including *CONSTITUTIVELY PHOTOMORPHOGENIC 1* (*COP1*), *SUPPRESSOR OF PHYA-105* (*SPA1*) and *HY5*, act as co-partners of photoreceptors or perception pigmentations in all three light signal transduction pathways [19, 28]. Among these unigenes, only *HY5* showed a significant decrease in response to shading treatment. *UVR8*, the specific photoreceptor of UV-B radiation identified in high plants to date, has been reported as constitutive expression and non light-induced in *A. thaliana* [41]. In this study, consistent results were found that the expression of *UVR8* did not change significantly in tea buds after shading. However, a unigene annotated as *UVR8 LIKE* (*UVR8_L*, TRINITY_DN27587_c4_g1_i3, 2222 bp) encoding a UVR8 like protein (XP_018821826) was observed to be significantly decreased at 4h and 8h of shading treatment in this study. Also, a significant circadian regulation was detected, with higher levels of *UVR8_L* transcript abundance at noon and relatively lower expression at late afternoon in tea buds. Other unigenes showed no significant change in response to shading in tea buds in this study.

Cluster analysis of these unigenes and TFs involved in flavonoid biosynthesis and different light signal transduction pathways revealed that these unigenes were differ in expression pattern in response to shading (Fig. 5c). Unigenes encoding main enzymes (*FLS*, *F3’H*, *ANS*, *ANR*, *LAR*, *DFR,* and *CHSs*) involved in the flavonoid biosynthetic pathway and TFs thought to regulate flavonoid biosynthesis (*MYB4*, *MYB12*, *MYB14,* and *MYB111*) presented consistent decreasing patterns in transcriptome datasets. A similar decreasing pattern was determined in potential unigenes and TFs from the UVR8-mediated signal transduction pathway (*UVR8_L*, *HY5*, *COP1*, and REPRESSOR OF UV-B PHOTOMORPHOGENESIS 1/2 (RUP1/2)), when compared with those involved in the UV-A/blue light and red/far-red light signal transduction pathways.

### Highly correlated gene expression patterns between flavonoid biosynthetic pathway and UV-B light signal transductions

**Fig. 6:**
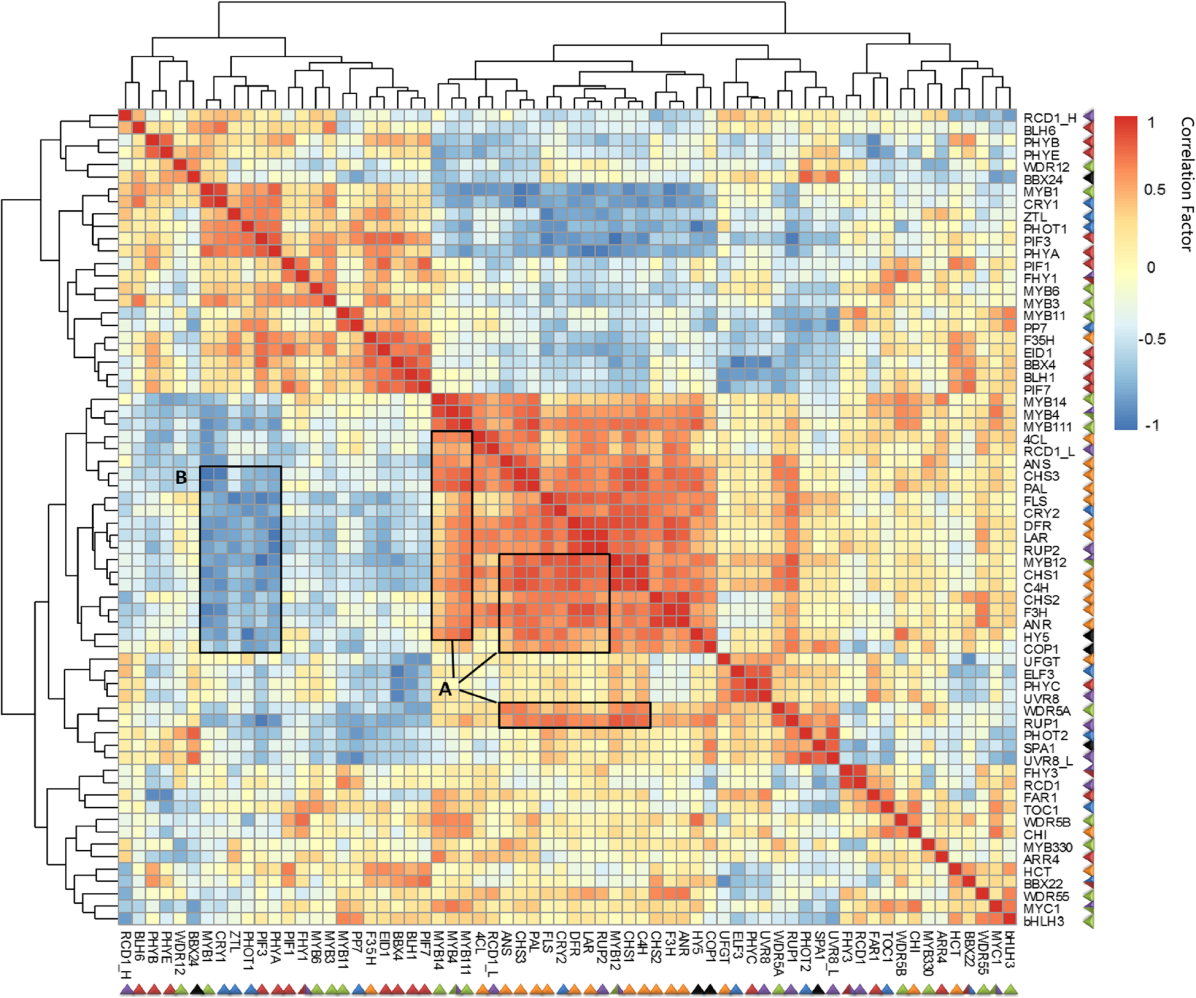
The matrix correlation of transcript abundance of potential genes and TFs involved in flavonoid biosynthesis and different light signal transduction pathways in response to shading treatment. The heatmap was conducted from the FPKM profiles of genes and TFs from transcriptome dataset in tea buds at five time points throughout shading treatments. Correlation factor indicates the correlation of transcriptional expression of two genes (− 1~0, expression of genes are negatively correlated; 0, expression of genes are not correlated; 0~1, expression of genes are positively correlated). Data were conducted from three biological replicates (n = 3), analysed by the Speaman test in SPSS 13.0 software (IBM SPSS Software, https://www.ibm.com/analytics/data-science/predictive-analytics/spss-statistical-software) and visualized by the “pheatmap” package implemented in R (https://cran.r-project.org/web/packages/pheatmap/index.html)

To further determine the correlation of these potential unigenes involved in flavonoid biosynthesis and different light signal transduction pathways at the transcriptional level, matrix correlation was conducted by a professional statistic analysis (Fig. 6). Matrix correlation analysis indicated that unigenes and TFs involved in the flavonoid biosynthetic pathway were positively correlated to potential unigenes involved in the UVR8-mediated signal transduction pathway in response to shading. As shown in Fig. 6a, many unigenes encoding enzymes involved in flavonol and catechin biosynthesis (*ANS*, *CHSs*, *PAL*, *FLS*, *DFR*, *LAR* and *C4H*, etc.) were positively correlated to annotated TFs (*MYB4*, *MYB12*, *MYB14* and *MYB111*, etc.). Meanwhile, these unigenes were also positively correlated to unigenes involved in the UVR8-mediated signal transduction pathway (*HY5*, *COP1*, *RUP1/2* and RADICAL-INDUCED CELL DEATH 1 (RCDs), etc.). *CRY1*, *CRY2* and *ZTL*, genes encoding photoreceptors of UV-A/blue lights showed some negative correlations in expression patterns with *CHSs* and *FLS* unigenes (Fig. 6b). However, no correlation was detected in the potential downstream signal mediators and TFs that involved in the UV-A/blue light signal transduction pathway. These data indicated the UV-B radiation might play a more intimate and crucial role in regulating flavonoid biosynthesis in tea plants in response to shading.

### Gene expression validated by quantitative real-time PCR (qRT-PCR)

**Fig. 7:**
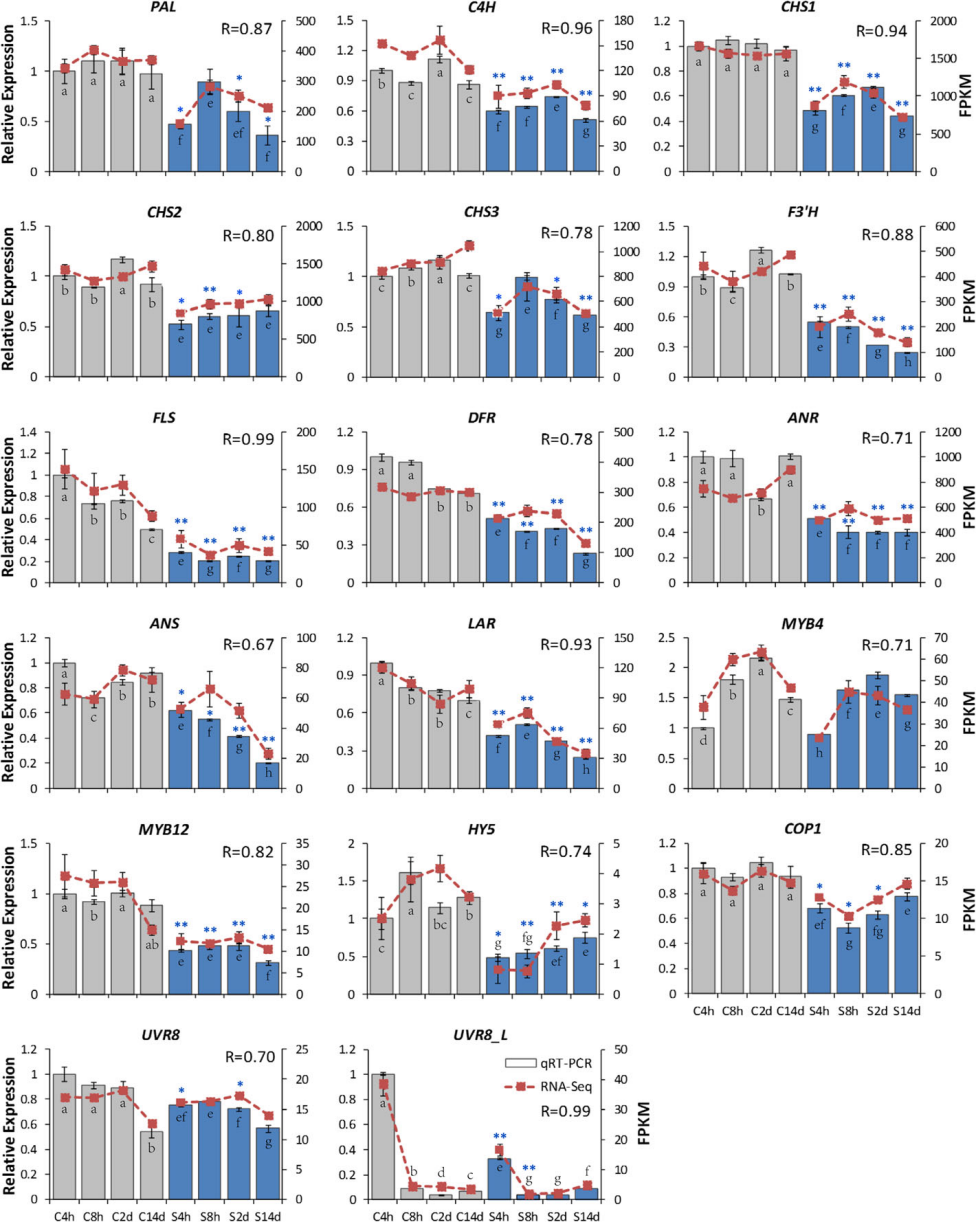
The shading effects on gene expression in tea buds analysed by both RNA-seq and qRT-PCR. R, relevance factor of gene expression between RNA-Seq and qRT-PCR data by the double factor correlation test in SPSS 13.0 software. C4h, C8h, C2d and C14d indicate samples collected at 4h, 8h, 2d and 14d in the Control treatment. S4h, S8h, S2d and S14d indicate samples collected at 4h, 8h, 2d and 14d in the shading treatment. Data shown are the average mean ± SE of three replicates (***n*** = 3). *Significant differences comparing the Control treatment at each time point according to one-way ANOVA and a Fisher’s LSD test at the 5% level (*p < 0.05, **p < 0.01). Different letters indicate statistical significance among time points for the Control (a, b, c, d) and Shading (e, f, g, h) treatments in qRT-PCR using one-way ANOVA and a Fisher’s LSD test at the 5% significance level

To validate and complete the data from our assembled tea transcriptome datasets, transcript abundance of 17 selected unigenes was analysed by qRT-PCR. These unigenes included 11 main structural genes of the flavonoid biosynthetic pathway (*PAL*, *C4H*, *CHSs*, *F3’H*, *FLS*, *DFR*, *ANR*, *ANS* and *LAR*), 2 MYB TFs involved in this pathway (*MYB4* and *MYB12*), and 4 components involved in the UVR8-mediated signal transduction pathway (*UVR8*, *UVR8_L*, *HY5* and *COP1*). Consistent shading responses were detected between the qRT-PCR analysis and RNA-Seq data (Fig. 7). Consistent with the transcriptome datasets, the expression of *CHSs*, *F3’H*, *FLS*, *DFR*, *ANR*, *ANS* and *LAR* significantly decreased in tea buds presented by qRT-PCR analysis, especially after 14 days of shading. *MYB12* showed a significant decrease in qRT-PCR data, which was consistent with the shading response found by RNA-Seq. Furthermore, a clear shading response was detected in *HY5*, with a significant decrease throughout the whole shading stages detected. *COP1* gave a slight but significant decrease, in particularly during earlier stages of shading. For UV-B photoreceptor, two unigenes were detected by qRT-PCR and the results were consistent with the RNA-Seq data. *UVR8* showed no significant shading response, which was consistent with the previous studies in *A. thaliana* that UVR8 is likely to be constitutively expressed and non light-induced [41, 42]. The unigene annotated as *UVR8_L* showed both shading responses and a circadian regulation.

### UV-B radiation activates flavonoid biosynthesis in controlled treatments

To complete the shading experiments in tea plantation and validate the UV-B effects on flavonoid biosynthesis in tea plants, a UV-B experiment was carried out in controlled environment using hydroponic tea plants. Tea plants were exposed to a supplementary of UV-B radiation in the controlled environment, with a similar UV-B fluence as in tea plantation (20 μW/cm^2^ of artificial fluence in the controlled environment; about 25 μW/cm^2^ of natural UV-B fluence in the clear summer afternoon). Both major catechins and genes involved in flavonoid biosynthesis and the UVR8-mediated signal transduction were measured (Fig. 2d and 9). EGCG showed some increase in tea buds in response to UV-B and GC gave a slight induction after 14 days of UV-B exposure (Fig. 9a). However, the other catechins measured in this study showed no significant responses. For genes measured in this study, *UVR8* and *COP1* showed no consistent UV-B response throughout the treatment (Fig. 9b). *HY5* expression presented a significant induction to UV-B exposure, with significant increases at 3d to 14d. The expression of *MYB12* increased significantly at 1d and 3d in response to UV-B, but no significant change was detected at later period. *FLS* gave a significant UV-B induction and the induction presented a similarly increasing pattern through time points as detected in *CHS1*. In contrast, *MYB4* showed a slightly increase after short period of UV-B exposure, then the expression decreased at 7d and 14d. Minor changes were detected for *C4H* and *LAR* expression but not consistent among time points.

## Discussion

There have been several studies showing the polyphenol accumulation can be changed in tea leaves under shading conditions, along with an enhancement of green color or pigmentation changes even in some yellow or purple phenotypes [10, 11, 43, 44]. Although the majority of previous studies have been carried out predominantly on the shading effects on catechin biosynthesis in different tea cultivars [11, 13, 44], this study is the first study to investigate the transcriptional regulation of flavonoid biosynthesis in relation to the shading responses delivered from different light signal transductions in *C. sinensis*.

### Shading stimulates chlorophyll accumulation

The accumulation of chlorophylls in tea buds was significantly modulated by light condition in tea plantation (Figs. 1, 2 and 3). Shading the tea plants by covering nylon nets, the green color of tea leaves was enhanced along with the simultaneous increases in chlorophyll accumulation (Fig. 1). These findings are consistent to the previous studies that the shaded leaf greening results from an increase in leaf chlorophyll and carotenoid abundance and chloroplast development [13, 44]. Besides the green tea phenotypes [11], similar shade-induced responses on chlorophylls and flavonoids were detected from yellow leaf phenotypes, such as albino cultivars *C. sinensis* cv. *Yujinxiang* [13] and *Baijiguan* [44]. It is suggested that pale yellow leaves in albino cultivars have aberrant chloroplast development, but shading treatment is able to recover (at least partially) normal leaf chloroplast development and leaf color [13]. However, there remains a great deal to be learnt about the regulatory metabolism of different light conditions leading to changes in chloroplast development and chlorophyll accumulation in tea plants.

### Shading represses the flavonoid biosynthetic pathway

The biosynthesis of flavonoids has been one of the most important areas with extensive studies in tea research [34, 45–48]. In the present study, flavonols and major catechins (in particular C, EC, GC and EGC) decreased significantly in tea buds that gave a different quantitative profile of flavonoid products throughout shading period (Fig. 3). Furthermore, the reduction of flavonoids was notably correlated with the period and the level of shading treatments. To explain the effects of shading on flavonoid biosynthesis in more detail, we studied relevant genes involved in the flavonoid biosynthetic pathway by both the RNA-Seq and qRT-PCR analyses. Of 16 known enzyme genes in the flavonoid biosynthetic pathway, most gave a significant shading response in tea buds (Fig. 5a and 7). *F3’H* and *FLS* significantly decreased throughout shading, whereas the others (*PAL*, *CHS*, *DFR*, *ANS, ANR* and *LAR*, etc.) temporally decreased in early or late shading stages. This is probably due to the different regulatory networks of individual gene activities in tea plants. These results were consistent to the previous findings on genes involved in the flavonoid biosynthetic pathway under shading conditions, particularly the *FLS* and *CHSs* genes in requirement for light exposure [13, 44]. The transcription of *FLS* and *F3’H* leading to quercetin and kaempferol have been reported to be decreased after shading which were positively correlated with the reduced accumulation of quercetin and kaempferol in albino cultivar *C. sinensis* cv. *Yujinxiang* [13]. Consistently, significant shading reduction of *FLS* expression was detected in this study with the simultaneous down regulation of keampferol-7-*O*-glucoside and kaempferol-3-*O*-galactopyranoside, but no quercetin was detected.

It’s still a mystery why tea plants accumulate such a high level of catechins (up to 25–30% of the dry weight of tea leaves) when compared with other high plants. It has been suggested that the catechins potentially serve as antioxidants to protect tea plants from oxidative stresses (including high light damage) and to promote stress tolerance in tea plants [29]. Recent analyses of transcriptome and phytochemistry data suggested that amplification and transcriptional divergence of genes encoding a large acyltransferase family and LARs are associated with the characteristic young leaf accumulation of monomeric galloylated catechins in tea plants [49]. The transcriptional regulation of the flavonoid biosynthetic pathway is mainly attributed to a MBW complex conducted of DNA-binding R2R3-MYB TFs, MYC-like basic helix-loop-helix and WD40 proteins [50–57]. However, due to the lack of genome information [49, 58], there has been no much detail on the transcription factors in relation to flavonoid biosynthesis and shading responses in tea plants. In this study, totally 26 unigenes of 15 TFs were annotated to be associated with flavonoid biosynthesis in our assembled transcriptome datasets, including *MYB4*, *MYB11*, *MYB12*, *MYB111* and *MYB330* (Additional file 3). TF MYB12 has been shown by transient reporter assays and complementation of *A. thaliana* mutants with flavonol-deficient phenotypes to be a positive regulator of *FLS* in grapevines [59]. Sequence analysis of *MYB12* and *FLS* suggested putative light regulatory units in the promoters of both genes [38]. In the present study, *MYB12* showed significantly lower expression in tea buds under shading condition, which was consistent with the decreasing pattern of *FLS* expression and flavonol accumulation (Fig. 3b, 5a and 7). These findings could suggest a regulatory role for *MYB12* in flavonol biosynthesis in *C. sinensis*, consistent to the previous findings from other high plants [37, 59]. In addition, a R2R3-MYB TF of tea plants named as CsMYB4a has been isolated and identified recently [34]. Transcriptional and metabolic analyses indicated *CsMYB4a* expression is negatively correlated to the accumulation of six flavan-3-ols and other phenolic acids. Further CsMYB4a-AC element and CsMYB4a-promoter interaction analyses suggested that the negative regulation of CsMYB4a on the flavonoid pathway is via reducing promoter activities of *CsC4H*, *Cs4CL*, *CsCHS*, *CsLAR* and *CsANR2* [34]. However, no significant shading effect was detected at the transcriptional level for *MYB4* or other TFs in this study, such as *MYB11* and *MYB111*.

### UVR8-mediated signaling genes are transcriptionally correlated with the flavonoid biosynthetic pathway

The understanding of molecular metabolism at the transcriptional level in tea plants has taken a step with the application of RNA-Seq technology [60–62]. Through our assembled tea transcriptome datasets, 46 representative unigenes (in total 77 annotated unigenes) thought to be involved in the UV-B, UV-A/blue and red/far-red light signal transductions were measured in this study (Additional files 4, 5 and 6). Cluster analysis of gene activities attributed the regulation of flavonoid accumulation to the UVR8-mediated signal transduction pathway (Fig. 5c). Indeed, the central signaling modulator *HY5* associated with plant photomorphogenesis and crossed over three light signal transduction pathways was significantly decreased in response to shading (Fig. 5b and 7). *MYB12*, the flavonol specific regulator, presented significant decrease after shading and clustered tightly to *HY5* and *FLS* expression in tea buds. Most of genes (*LAR*, *F3’H*, *ANS*, *ANR*, *DFR*, *CHI* and *CHSs*, etc.) and TFs (*MYB4*, *MYB14*, *MYB111* and *WDR5A*) associated with the flavonoid biosynthetic pathway showed similar responses to the signal regulators involved in the UVR8-mediated signal transduction pathway (*COP1* and *RUP1/2*). According to the previous studies in *A. thaliana*, *MYB11*, *MYB12* and *MYB111* activate, in parallel, the biosynthetic enzyme encoding genes *CHS*, *CHI*, *F3H* and *FLS* leading to flavonol accumulation [35, 36]. These MYB TFs were also found to control the spatial distribution of flavonoids at the transcriptional level [36, 38] and their functions were light/UV-B inducible [33, 63]. Furthermore, *MYB4* has been found in tea plants to be a transcriptional repressor of several key enzyme encoding genes involved in flavonol and catechin biosynthesis [34]. *MYB12* has been shown to be light inducible and positively regulating the activity of *FLS* in response to light, specifically UV-B radiation in plants [30, 37]. The involvements and characteristics of UV-induced TFs, together with their similar responses to shading implied that the UVR8-mediated signal transduction pathway played a more central role in modulating flavonoid biosynthesis in tea plants, when compared to the UV-A/blue and red/far light signal transduction pathways.

This view was supported by KEGG enrichment and further matrix correlation analysis (Figs. 4 and 6). KEGG enrichment indicated that genes involved in the UVR8-mediated signal transduction pathway were significantly affected by shading and potentially played a more crucial role in regulating flavonoid biosynthesis in response to shading than other light signal pathways. In addition to KEGG enrichment, the matrix correlation analysis showed that main genes (*ANS*, *CHSs*, *PAL*, *FLS*, *DFR*, *LAR* and *C4H*, etc.) and TFs (*MYB4, MYB12*, *MYB14* and *MYB111*, etc.) associated with the flavonoid biosynthetic pathway presented a clear and positive correlation to potential genes involved in the UVR8-mediated signal transduction pathway (*HY5*, *COP1*, *RUP1/2* and *RCDs*, etc.; Fig. 6a). Photoreceptors, including specific pigmentations and proteins involved in UV-A/blue light perceptions, showed some negative correlations to structural genes of the flavonoid biosynthetic pathway (*CRY1/2* and *ZTL; CHSs* and *FLS* genes). However, no correlation was found for the downstream genes and TFs between the flavonoid biosynthetic pathway and UV-A/blue light transduction pathway (Fig. 6b).

### UV-B signal transduction pathway mediates the shading-reduced flavonoid biosynthesis

Shading usually leads to changes in plant morphogenesis and activations of the shade-avoidance syndrome [16–18]. Previous studies have indicated that the composition of light wavelengths can be modified under shading conditions, with UV-B radiation significantly attenuated when compared with the natural sunlight [16, 18]. It has been suggested that UVR8-mediated plant responses are likely involved in morphological changes due to the shading-caused UV-B strength attenuation and dosage reduction [16]. We observed chlorophyll increases in tea leaves and delayed budding of young shoots under shading conditions in this study (Fig. 1b and c). Our study also showed that the shading effects included quantitative changes in flavonol and catechin accumulation (Fig. 3), modifications in transcript abundance of genes and TFs involved in flavonoid biosynthesis (Fig. 5a and 7), as well as responses in activities of potential genes and mediators associated with different light signal transductions in tea buds (Fig. 5b and 7). Further, transcriptome data revealed the co-expression of these light signaling genes with flavonoid biosynthesis- and regulation-related genes and TFs (Fig. 6a).

**Fig. 8:**
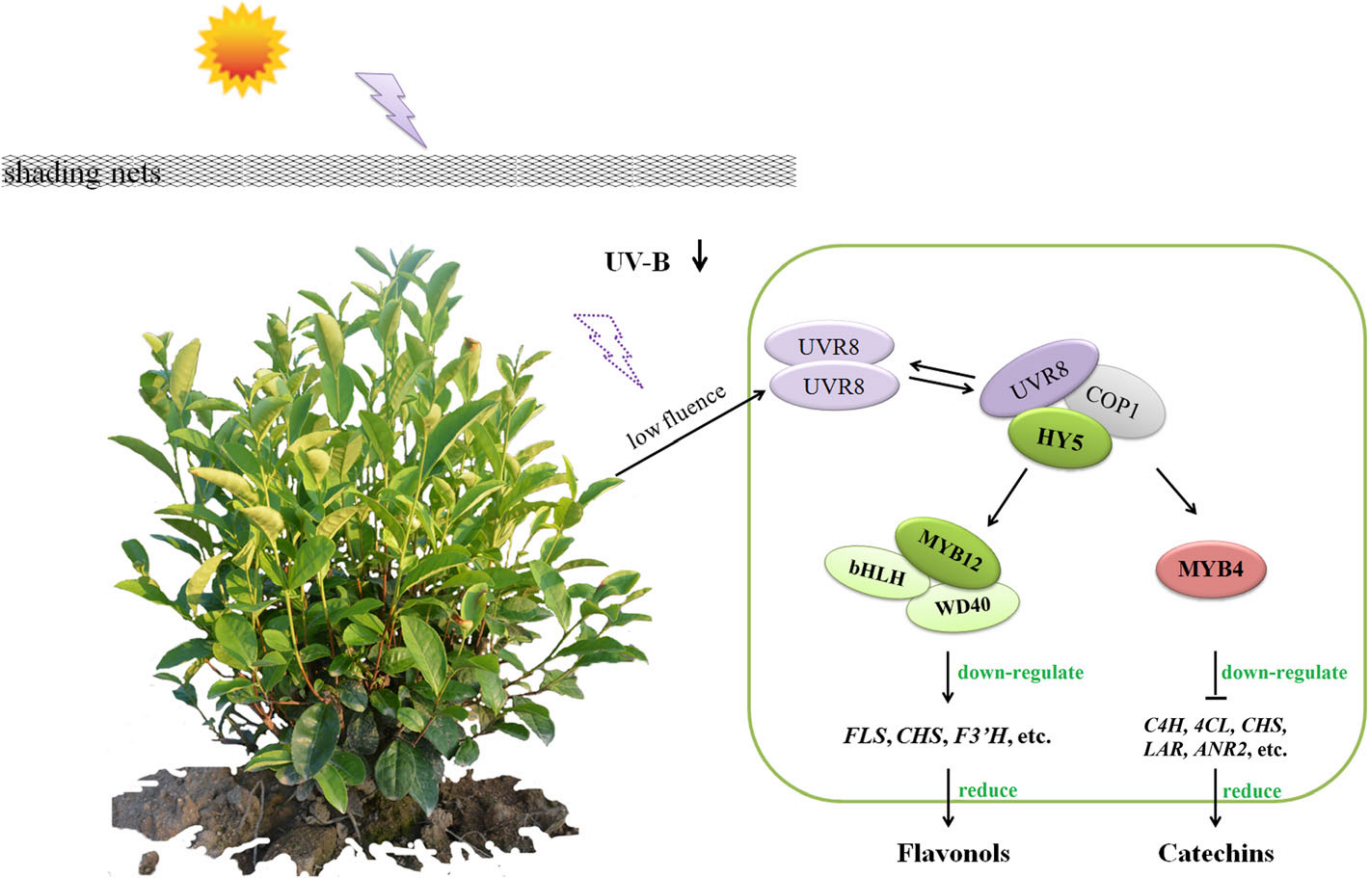
Working model for flavonoid biosynthesis in tea plants regulated by the UVR8-mediated signal transduction pathway in response to shading conditions. The full names of genes and TFs are shown in abbreviation

As modeled in Fig. 8, the photoreceptions and early signal delivery of the UVR8-mediated low fluence UV-B responses have been largely characterized in model plant *A. thaliana* [25, 26, 39]. Under visible light (400–750 nm), UVR8 proteins in plant cells appear as homodimers. Once UV-B signal is perceived by the intrinsic tryptophan chromophores, UVR8 homodimer dissociates into their active monomer conformation [64, 65]. The activated UVR8 monomer interacts directly with a positive regulator COP1 leading to HY5 stabilization and enhances binding of HY5 to the promoter regions of UV-B responsive genes [27, 31, 66]. TF MYB12, a UV-B inducible and specific positive regulator for *FLS* [30, 37, 59], together with TF MYB4 which functions as a transcriptional repressor of *LAR* and *ANR2*, etc. [34] delivers the UV-B responses from early UVR8-mediated signal events to relevant genes in the flavonoid biosynthetic pathway and eventually modulates the flavonol and catechin accumulation in plants [32–34]. Other R2R3-type MYB TFs, including MYB11, MYB14 and MYB111 which have been reported to control the expression of *CHS*, *CHI*, *F3’H* and *FLS1* are likely to be involved in this regulatory mechanism [35, 36]. However when emerging underneath an established canopy or shading condition, the dissociation of UVR8 homodimers becomes constrained, either through degradation or an unknown negative regulation step due to UV-B attenuation [40], thus leads to a reduction of HY5 stabilization. This response limits promotion of *MYB12* and reduces activation of downstream responsive genes in the flavonoid biosynthetic pathway (*FLS*, *CHSs*, and *F3’H*, etc.), therefore cutoffs the investment in final products, flavonols, etc. Meanwhile, the reduction of UV-B radiation might down regulate the expression of relevant genes leading to flavonols (*C4H*, *4CL* and *CHS*, etc.) and catechins (*LAR* and *ANR*, etc.) through the regulation (activation) of MYB4, eventually operates the flavonol and catechin production in response to shading condition in tea plants.

**Fig. 9:**
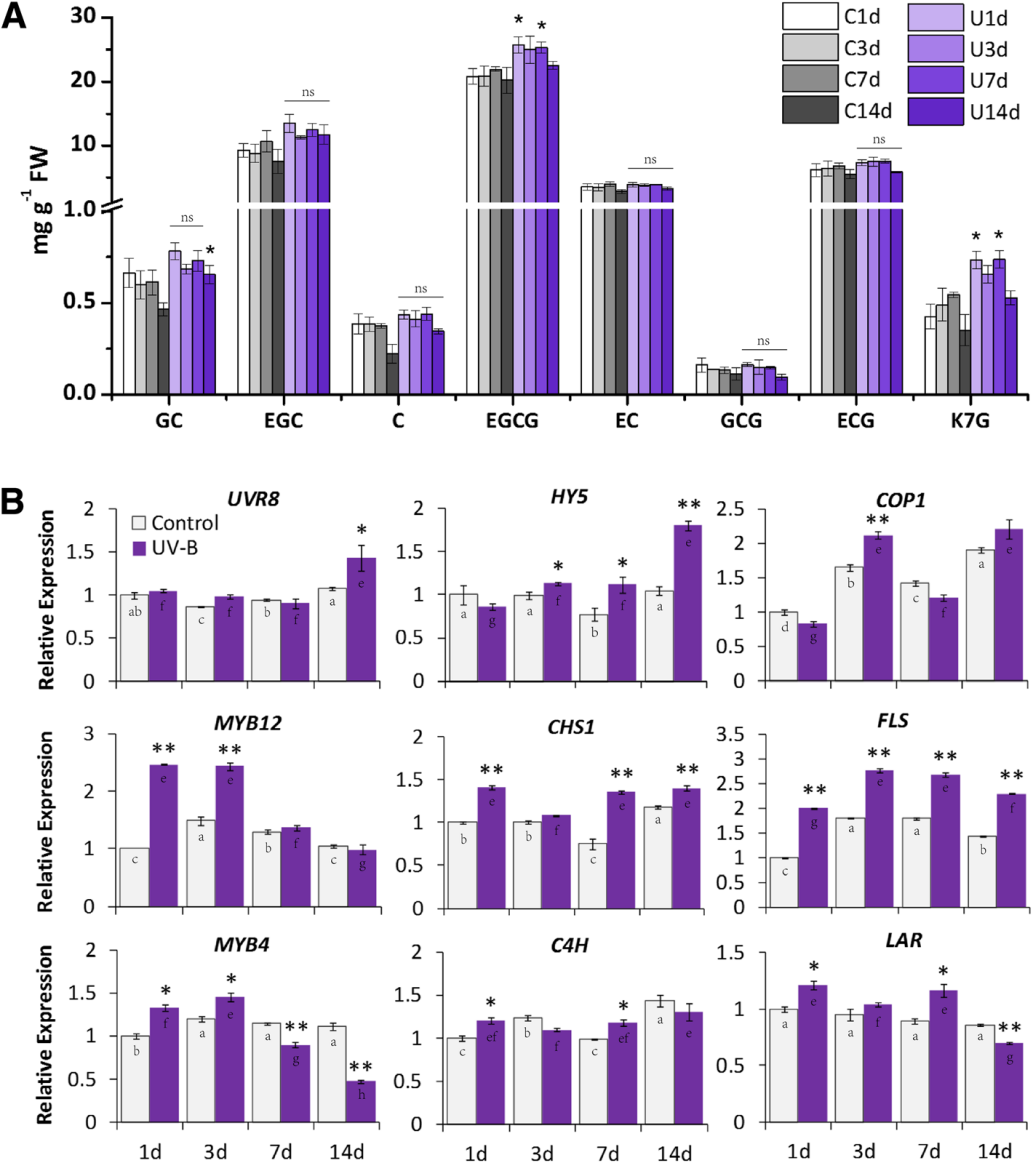
The effects of UV-B on major catechins and gene expression in UV-B experiment carried out in the controlled environment. **a** The concentration of major catechins in tea sample (bud with one developing leaf) from both the control and UV-B treatments in the controlled environment analysed by HPLC. **b** The expression of annotated unigenes in tea buds from both the Control and UV-B treatments in the controlled environment analysed by qRT-PCR. The full name of catechins and description of treatments in the controlled environment UV-B experiment are shown as above in Fig. 2. C1d, C3d, C7d and C14d indicate samples collected at 1d, 3d, 7d and 14d in the Control treatment. U1d, U3d, U7d and U14d indicate samples collected at 1d, 3d, 7d and 14d in the UV-B treatment. Data shown are the average mean ± SE of three replicates (n = 3). ns, no significance. *Significant differences comparing the Control treatment at each time point according to one-way ANOVA and a Fisher’s LSD test at the 5% level (*p < 0.05, **p < 0.01). Different letters indicate statistical significance among time points for the Control (a, b, c, d) and UV-B (e, f, g, h) treatments using one-way ANOVA and a Fisher’s LSD test at the 5% significance level

To further validate the effects of UVR8-meditated signal transduction pathway in regulating flavonoid biosynthesis in tea plants, a controlled environment experiment was conducted using hydroponic tea plants and artificial supplement of UV-B radiation (Fig. 9). In contrast to reduction of flavonols and catechins in tea buds under shading condition, flavonols and catechins showed some increases after UV-B exposure in the controlled environment (Fig. 9a). This is particularly interesting in respect to a previous publication suggesting that low fluence and short term of UV-B radiation stimulates the accumulation of major tea catechins including GC, EC and EGCG, finally results in an increase of total catechins in tea leaves [29]. Consistent results were detected in this study, with both GC and EGCG abundance presented some increases after UV-B exposure. The fluence of UV-B radiation plays an important role in determining gene expression and flavonoid biosynthesis, either through the specific UV-B signal transduction pathway mediated by UVR8 or high fluence UV-B being recognized as a potential damaging/stress signal and lead to many known signal transduction mechanisms including pathogen-related proteins and reactive oxygen species [26, 39, 51]. Furthermore, significant increases detected in transcriptional expression of *HY5*, *MYB12*, *FLS* and *CHS1* suggest a central role of *MYB12* in delivering UV-B responses from early UV-B perception to downstream responsive genes in flavonoid biosynthesis (Fig. 9b). This is consistent with the function of *MYB12* in regulating flavonols under UV-B radiation in other commercially important plants, such as grapevine and apple [51, 52, 54]. In addition, significant changes were also found in *MYB4* expression under controlled condition, with slight increases after short term of UV-B exposure (1d and 3d) and decreases after long term of UV-B exposure (7d and 14d, Fig. 9b). The UV-B induced decreases in *MYB4* expression is consistent with the previous findings that *MYB4* is down regulated in response to UV-B radiation [32, 33]. Together, the UV-B responses of flavonols and catechins, the transcriptional changes of candidate genes and TFs involved in flavonoid biosynthesis and the UVR8-mediated signal transduction pathway, both suggest an essential role of UV-B radiation in modulating flavonoid biosynthesis in tea plants.

## Conclusions

Flavonoid biosynthesis in tea plants is tightly regulated by both internal regulatory factors and environmental cues. Especially, light signal consisted of various wavelengths including UV-B radiation is the crucial regulator for flavonoid biosynthesis in tea plantation. We here demonstrated that the reduction of flavonols and catechins in shading tea plants was mainly modulated through the down regulation of biosynthetic genes and TFs associated with flavonoid biosynthesis caused by reduced UV-B radiation. When compared with the UV-A/blue and red/far-red light signal transductions, genes involved in the UVR8-mediated signal transduction pathway are more central to determining flavonoid production in tea plants in response to shading. This study provides new insights into our understanding of shading effects on flavonoid biosynthesis in tea plants, which has been an often used strategy to improve tea quality. The UV-B signal transduction is identified as the key factor to regulate flavonoid production in response to shading in a non-model and important commercial plant, *C. sinensis*.

## Methods

### Shading treatments in tea plantation

The shading experiment was carried out in Anhui Agricultural University research tea plantation (31°. 55’ North, 117°. 12′ East; Hefei City, Anhui Province, China). 12 rows of *C. sinensis* cv. *Shuchazao* tea plants (50 m long and 1.4 m wide of each row, 2 m between and 0.6 m within row spacing) were selected for the treatments. The tea plants were 24 years old from cuttage propagation (1.4 m wide and 1.5 m tall from the soil surface, 0.5 m between plants within the row). The set up of shading treatments are shown as in Fig. 1a. The nylon black nets with different light transmitting characteristics (Nongfeng Company, Hefei, China) were placed about 1.5 m over the tea plants for shading treatments. Each treatment was replicated three times and the positions of treatments randomized statistically within the rows. The shading experiment was consisted of three treatments: tea plants with naturally growth (Control); tea plants with 50–60% shading treatment (S50–60%, 40–50% of natural sunlight can be transmitted through the nets); tea plants with 80–90% shading treatment (S80–90%, 10–20% of natural sunlight can be transmitted). The nets were placed over the plants on 12^nd^ of April in 2017 when a new round of bud burst started. Tea buds were collected throughout shading treatments (4h, 8h, 2d, 4d, 8d, and 14d after shading). All the materials were frozen immediately in the field using liquid nitrogen and stored at − 80 °C for future use.

Environmental parameters were measured among each treatment to monitor the growth conditions of tea plants during shading experiment, including the PAR (Light Scout® Quantum Light Meters, Item#3415F, Spectrum Technology® Inc. USA), temperature, humidity, and the content of CO_2_ around tea leaves (TEMP/RH/CO_2_ hand-held meter, catalog#3440, Spectrum Technology® Inc. USA).

### UV-B treatment under the controlled condition

A controlled environment cabinet with full PAR transmission was used for UV-B treatment (3 m wide and 5 m length). *C. sinensis* cv. *Shuchazao* tea plants were grown from cuttage propagation in Anhui Agricultural University research tea plantation for two years. Before the UV-B treatment started, tea plants were moved into the cabinet and grown in a hydroponic culture system [67] for about three months until the new root and buds began to grow. Tea plants with good growth conditions and similar size were selected and divided into two groups: 60 plants for the UV-B treatment (UV-B), 60 plants for the control treatment (Control). Tea plants for UV-B treatment were exposed to PAR and supplementary UV-B radiation (20 μW/cm^2^, 8 h/d), while the control plants were exposed to pure PAR. Samples were collected at 1d, 3d, 7d, and 14d from both the UV-B and Control treatments. At each time point, 15 plants from the Control treatment and 15 plants from the UV-B treatment were removed from the cabinet. Tea bud with one developing leaf were immediately collected and frozen in liquid nitrogen (5 plants for each biological replicate), then stored at − 80 °C for future use.

UV-B radiation was provided by UVB-313 UV fluorescent tubes (Q-Lab Company, Westlake, USA). UV-B Fluence was measured by a UVB Biometer model 501 radiometer (Solar Light Company, Glenside, PA, USA). Temperature (24 °C /18 °C, day/night) and humidity (70–80%) were controlled in the cabinet.

### Chlorophyll analysis

0.1 g fresh tea buds were cut into small pieces. Chlorophylls were extracted overnight using 10 mL solvent (5% acetone in 95% ethanol, *v*/v) until the pieces became completely white, then the extraction was measured using an ultraviolet spectrophotometer (U-5100, Hitachi, Japan) at A_645_ and A_663_. The chlorophyll contents were calculated using the following formula: Chl_a_ = 12.70 A_663_–2.69 A_645_; Chl_b_ = 22.9A_645_–4.68A_663_.

### Flavonoid analysis

Tea flavonoids were extracted and measured according to the methods previously described with minor modifications [62]. Frozen tea buds were grounded in liquid nitrogen with a mortar and pestle. 0.1 g of the sample was extracted with 3 mL 80% methanol in an ultrasonic sonicator for 10 min at 4 °C. After centrifugation (13,000 rpm, 10 min), the residues were re-extracted twice as described above. The supernatants were combined and diluted with 80% methanol to a fixed volume of 10 mL. Then the supernatants were filtered through a 0.22 μm organic membrane and collected for HPLC analysis.

Tea flavonoids were measured using a HPLC system (Waters 2695) coupled to an ultraviolet-visible detector (Waters 2489) as previously described with modifications [62]. A reverse phase C18 column (Phenomenex 250 mm × 4.6 mm, 5 μm) was used at a flow rate of 1.0 mL min^− 1^. The detection wavelength was set at 278 nm at a column temperature of 25 °C. The separation used solvent A (0.2% acetic acid in water, v/v) and solvent B (100% methanol) with the following gradient: 0 min, 94% A, 6% B; 4 min, 94% A, 6% B; 16 min, 86% A, 14% B; 22 min, 85% A, 15% B; 32 min, 82% A, 18% B; 37 min, 71% A, 29% B; 45 min, 55% A, 45% B; 50 min, 55% A, 45% B; 51 min, 94% A, 6% B; 60 min, 94% A, 6% B. 10 μL of the extraction was injected for analysis. C, EC, GC, EGC, EGCG, GCG, ECG, K3G and K7Gal were used as standards for sample quantification (Sigma Chemical Company, St. Louis, MO, USA). The total catechin contents were calculated as the sum of seven individual catechins.

### RNA-Seq analysis

The high-quality RNA extraction for the Control and S80–90% treatments, library conduction and RNA-Seq performed by the Illumina HiSeq2000 were carried out professionally in Wuhan Bosaixi Biotechnology Company (Wuhan, China). Clean reads were combined and assembled separately using the transcriptome assembler Trinity (version r20140717) with default parameters [68]. Unigene were functional annotated with alignments to the NR, Swiss-Prot, KOG, KEGG and GO databases, respectively.

The KEGG enrichment analysis was conducted according to the DEGs in the assembled tea transcriptome datasets and KEGG database visualized in R (https://cran.r-project.org/web/packages/pheatmap/index.html). The heatmaps for gene expression were conducted using the “pheatmap” package implemented in R. The competitive expression (the thresholds of log_2_ FPKM_shading_/FPKM_control_) was used to conduct heatmaps for gene expression. The matrix correlation of transcript abundance (FPKM values) among genes and TFs associated with the flavonoid biosynthesis and different light signal transduction pathways were analysed by the Speaman test in SPSS 13.0 software (IBM SPSS Software, https://www.ibm.com/analytics/data-science/predictive-analytics/spss-statistical-software) and visualized by the “pheatmap” package implemented in R.

### qRT-PCR analysis

To validate the accuracy of unigenes obtained from the assembled tea transcriptome datasets and profiling of gene expression via RNA-Seq, qRT-PCR was performed for the selected unigenes. Total RNA was isolated from tea buds using the Spectrum™ Plant Total RNA Kit (Sigma-Aldrich, Shanghai, China). RNA samples were treated by the TURBO DNA-free™ Kit (Sigma-Aldrich, Shanghai, China) to remove traces of genomic DNA. Single-stranded cDNAs used for qRT-PCR were synthesized using a Prime-Script™ Strand cDNA Synthesis Kit (TaKaRa, Dalian, China). qRT-PCR was carried out using the SYBR green method for detection of double-stranded PCR products (TaKaRa, Dalian, China). An IQ5 real-time PCR detection system (Bio-Rad) was utilized in this study as previously described [62]. The tea *β-actin* gene was used as an internal reference gene (HQ420251.1, https://www.ncbi.nlm.nih.gov/nuccore/HQ420251.1) [69]. The primers for 17 selected unigene in this study were designed by Primer Premier 5.0 software (PREMIER Biosoft Company, http://www.premierbiosoft.com/index.html, Additional file 7).

### Statistic analysis

All the data presented in this study were calculated from three independent biological replicates, including chlorophylls, the total and individual catechins/flavonols, RNA-Seq and qRT-PCR analysis. Statistical analyses were conducted using the Minitab 16.0 statistical software package (Minitab Inc., Coventry, UK). Data were analysed by one-way analysis of variance (ANOVA) and a Fisher’s least significant difference (LSD) test at the 5% level.

## Additional files

Additional file 1: Primers of annotated unigenes designed for qRT-PCR analysis in this study. (XLSX 12 kb)

Additional file 2: Statistics information from the generated transcriptome reads. (XLSX 16 kb)

Additional file 3: Transcript abundance of main genes involved in the flavonoid biosynthetic pathway annotated in tea transcriptome datasets. (XLSX 12 kb)

Additional file 4: Transcript abundance of potential TFs involved in the flavonoid biosynthetic pathway annotated in tea transcriptome datasets. (XLSX 12 kb)

Additional file 5: Transcript abundance of potential genes involved in the UVR8-mediated signal transduction pathway annotated in tea transcriptome datasets. (XLSX 11 kb)

Additional file 6: Transcript abundance of potential genes involved in the UV-A/blue light signal transduction pathway annotated in tea transcriptome datasets. (XLSX 12 kb)

Additional file 7: Transcript abundance of potential genes involved in the red/far-red light signal transduction pathway annotated in tea transcriptome datasets. (XLSX 10 kb)

## Abbreviations

4Cl: 4-coumarate-CoA ligase
ANR: Anthocyanidin reductase
ANS: Anthocyanidin synthase
ARR4: ARABIDOPSIS RESPONSE REGULATOR 4
BBX4/22/24: B-BOX DOMAIN PROTEIN 4/22/24
bHLH: Basic Helix-Loop-Helix transcription factor
BLH1/6: BELLLIKE HOMEODOMAIN 1/6
C: Catechin
C4H: Cinnamate 4-hydroxylase
CHI: Chalcone isomerase
CHS: Chalcone synthase
COP1: CONSTITUTIVELY PHOTOMORPHOGENIC 1
CRY1/2: Cryptochrome 1/2
DFR: Dihydroflavonol-4-reductase
EC: Epicatechin
ECG: Epicatechin gallate
EGC: Epigallocatechin
EGCG: Epigallocatechin gallate
EID1: EMPFINDLICHER IM DUNKELROTEN LICHT1
ELF3: Early flowering 3
F3’5’H: Flavonoid 3′,5′-hydroxylase
F3’H: Flavanone 3-hydroxylase
FAR1: FAR-RED-IMPAIRED RESPONSE1
FHY1/3/6: FAR-RED ELONGATED HYPOCOTYL 3/6
FLS: Flavonol synthase
FPKM: Fragment Per Kilo base of exon model per Million mapped reads
GC: Gallocatechin
GCG: Gallocatechin gallate
HCT: Hydroxycinnamoyl transferase
HPLC: High-performance liquid chromatographic
HY5: ELONGATED HYPOCOTYL 5
K3Gal: Kaempferol-3-*O*-galactopyranoside
K7G: Keampferol-7-*O*-glucoside
LAR: Leucoanthocyanidin reductase
MBW: MYB-bHLH-WD40 complex
MYC: Basic Helix-Loop-Helix transcription factor MYC1
PAL: Phenylalanine ammonialyase
PAR: Photosynthetically active radiation
PHOT1/2: Phototropin 1/2
PHYA/B/C/D/E: Phytochrome A/B/C/D/E
PIF1/3/7: PHYTOCHROME INTERACTING FACTOR 1/3/7
PP7: Ser/Thr protein phosphatise 7
qRT-PCR: Quantitative real-time PCR
RCD1: RADICAL-INDUCED CELL DEATH 1
RUP1/2: REPRESSOR OF UV-B PHOTOMORPHOGENESIS 1/2
SPA1: SUPPRESSOR OF PHYA-105
TF: Transcription factor
TOC1: TIMING OF CAB EXPRESSION1
UFGT: UDP-glucose flavonoid 3-O-glucosyltransferase
UV-B: Ultraviolet-B radiation
UVR8: UV RESISTANCE LOCUS8
UVR8_L: UV RESISTANCE LOCUS8 LIKE
WDR: WD40 repeat protein
ZTL: ZEITLUPE

## References

[1] Banerjee B. Botanical classification of tea. In: Willson KC, Clifford MN (eds). Tea: Springer Dordrecht Netherlands; 1992. p. 1133–4.

[2] Chacko SM, Thambi PT, Kuttan R, Nishigaki I (2010). Beneficial effects of green tea: a literature review. Chin Med-UK.

[3] Rogers PJ, Smith JE, Heatherley SV, Pleydellpearce CW (2008). Time for tea: mood, blood pressure and cognitive performance effects of caffeine and theanine administered alone and together. Psychopharmacology.

[4] Wang D, Wei Y, Wang T, Wan X, Yang CS, Reiter RJ, Zhang J (2015). Melatonin attenuates (−)-epigallocatehin-3-gallate-triggered hepatotoxicity without compromising its downregulation of hepatic gluconeogenic and lipogenic genes in mice. J Pineal Res.

[5] Hara Y, Jain NK, Rahman F, Baker P (2004). Health benefits and industrial applications of tea catechins. Int J Tea Sci.

[6] Scharbert S, Hofmann T (2005). Molecular definition of black tea taste by means of quantitative studies, taste reconstitution, and omission experiments. J Agr Food Chem..

[7] Rossetti D, Bongaerts JHH, Wantling E, Stokes JR, Williamson AM (2009). Astringency of tea catechins: more than an oral lubrication tactile percept. Food Hydrocolloid.

[8] Kaneko S, Kenji K, Hideki M, Andrea H, Hofmann T (2006). Molecular and sensory studies on the umami taste of Japanese green tea. J Agr Food Chem.

[9] Feng L, Gao MJ, Hou RY, Hu XY, Zhang L, Wan XC, Wei S (2014). Determination of quality constituents in the young leaves of albino tea cultivars. Food Chem.

[10] Song L, Ma Q, Zou Z, Sun K, Yao Y, Tao J, Kaleri NA, Li X (2017). Molecular link between leaf coloration and gene expression of flavonoid and carotenoid biosynthesis in *Camellia sinensis* cultivar ‘Huangjinya’. Front Plant Sci.

[11] Wang YS, Gao LP, Shan Y, Liu YJ, Tian YW, Xia T (2012). Influence of shade on flavonoid biosynthesis in tea (*Camellia sinensis* (L.) O. Kuntze). Sci Hortic-Amsterdam.

[12] Song R, Kelman D, Johns KL, Wright AD (2012). Correlation between leaf age, shade levels, and characteristic beneficial natural constituents of tea (*Camellia sinensis*) grown in Hawaii. Food Chem.

[13] Liu GF, Han ZX, Feng L, Gao LP, Gao MJ, Gruber MY, Zhang ZL, Xia T, Wan XC, Wei S (2017). Metabolic flux redirection and transcriptomic reprogramming in the albino tea cultivar ‘Yu-Jin-Xiang’ with an emphasis on catechin production. Sci Rep-UK.

[14] Zhang Q, Shi Y, Ma L, Yi X, Ruan J (2014). Metabolomic analysis using ultra-performance liquid chromatography-quadrupole-time of flight mass spectrometry (UPLC-Q-TOF MS) uncovers the effects of light intensity and temperature under shading treatments on the metabolites in tea. PLoS One.

[15] Pacín M, Semmoloni M, Legris M, Finlayson SA, Casal JJ (2016). Convergence of CONSTITUTIVE PHOTOMORPHOGENESIS 1 and PHYTOCHROME INTERACTING FACTOR signalling during shade avoidance. New Phytol.

[16] Ballaré CL (2014). Light regulation of plant defense. Annu Rev Plant Biol.

[17] Gelderen VK, Kang C, Paalman R, Keuskamp DH, Hayes S, Pierik R (2018). Far-red light detection in the shoot regulates lateral root development through the HY5 transcription factor. Plant Cell.

[18] Ballaré CL, Scopel AL, Sánchez RA (1990). Far-red radiation reflected from adjacent leaves: an early signal of competition in plant canopies. Science.

[19] Galvão VC, Fankhauser C (2015). Sensing the light environment in plants: photoreceptors and early signaling steps. Curr Opin Neurobiol.

[20] Burgie ES, Vierstra RD (2014). Phytochromes: an atomic perspective on photoactivation and signaling. Plant Cell.

[21] Ballaré CL (2009). Illuminated behaviour: phytochrome as a key regulator of light foraging and plant anti-herbivore defence. Plant Cell Environ.

[22] Casal JJ, Candia AN, Sellaro R (2014). Light perception and signalling by phytochrome a. J Exp Bot.

[23] Chaves I, Pokorny R, Byrdin M, Hoang N, Ritz T, Brettel K, Essen LO, Gt VDH, Batschauer A, Ahmad M (2011). The cryptochromes: blue light photoreceptors in plants and animals. Annu Rev Plant Biol.

[24] Suetsugu N, Wada M (2013). Evolution of three LOV blue light receptor families in green plants and photosynthetic stramenopiles: phototropin, ZTL/FKF1/LKP2 and aureochrome. Plant Cell Physiol.

[25] Jenkins GI (2014). The UV-B photoreceptor UVR8: from structure to physiology. Plant Cell.

[26] Yin R, Ulm R (2017). How plants cope with UV-B: from perception to response. Curr Opin Plant Biol.

[27] Rizzini L, Ulm R (2011). Perception of UV-B by the Arabidopsis UVR8 protein. Science.

[28] Kami C, Lorrain S, Hornitschek P, Fankhauser C (2010). Light-regulated plant growth and development. Curr Top Dev Biol.

[29] Zheng XQ, Jin J, Chen H, Du YY, Ye JH, Lu JL, Lin C, Dong JJ, Sun QL, Wu LY (2008). Effect of ultraviolet-B irradiation on accumulation of catechins in tea *Camellia sinensis* (L) O. Kuntze. Afr J Biotechnol.

[30] Stracke R, Favory JJ, Gruber H, Bartelniewoehner L, Bartels S, Binkert M, Funk M, Weisshaar B, Ulm R (2010). The Arabidopsis bZIP transcription factor HY5 regulates expression of the PFG1/MYB12 gene in response to light and ultraviolet-B radiation. Plant Cell Environ.

[31] Brown BA, Jenkins GI (2008). UV-B signaling pathways with different fluence-rate response profiles are distinguished in mature Arabidopsis leaf tissue by requirement for UVR8, HY5, and HYH. Plant Physiol.

[32] Schenke D, Böttcher C, Scheel D (2011). Crosstalk between abiotic UV-B stress and biotic (flg22) stress signaling in Arabidopsis prevents flavonol accumulation in favor of pathogen defense compound production. Plant Cell Environ.

[33] Zhang L, Wang Y, Sun M, Wang J, Kawabata S, Li Y (2014). *BrMYB4*, a suppressor of genes for phenylpropanoid and anthocyanin biosynthesis, is downregulated by UV-B but not by pigment-inducing sunlight in turnip cv. Tsuda. Plant Cell Physiol..

[34] Li M, Li Y, Guo L, Gong N, Pang Y, Jiang W, Liu Y, Jiang X, Zhao L, Wang Y (2017). Functional characterization of tea (*Camellia sinensis*) MYB4a transcription factor using an integrative approach. Front Plant Sci.

[35] Stracke R, Jahns O, Keck M, Tohge T, Niehaus K, Fernie AR, Weisshaar B (2010). Analysis of PRODUCTION OF FLAVONOL GLYCOSIDES-dependent flavonol glycoside accumulation in *Arabidopsis thaliana* plants reveals MYB11-, MYB12- and MYB111-independent flavonol glycoside accumulation. New Phytol.

[36] Stracke R, Ishihara H, Huep G, Barsch A, Mehrtens F, Niehaus K, Weisshaar B (2007). Differential regulation of closely related R2R3-MYB transcription factors controls flavonol accumulation in different parts of the *Arabidopsis thaliana* seedling. Plant J.

[37] Mehrtens F, Kranz H, Bednarek P, Weisshaar B (2005). The Arabidopsis transcription factor MYB12 is a flavonol-specific regulator of phenylpropanoid biosynthesis. Plant Physiol.

[38] Hartmann U, Sagasser M, Mehrtens F, Stracke R, Weisshaar B (2005). Differential combinatorial interactions of cis-acting elements recognized by R2R3-MYB, BZIP, and BHLH factors control light-responsive and tissue-specific activation of phenylpropanoid biosynthesis genes. Plant Mol Biol.

[39] Tilbrook K, Arongaus AB, Binkert M, Heijde M, Yin R, Ulm R (2013). The UVR8 UV-B photoreceptor: perception, signaling and response. Arabidopsis Book.

[40] Lau OS, Deng XW (2012). The photomorphogenic repressors COP1 and DET1: 20 years later. Trends Plant Sci.

[41] Heijde M, Binkert M, Yin R, Aresorpel F, Rizzini L, Van EDS, Persiau G, Nolf J, Gevaert K, De GJ (2013). Constitutively active UVR8 photoreceptor variant in Arabidopsis. P Natl Acad Sci USA..

[42] Heilmann M, Jenkins GI (2013). Rapid reversion from monomer to dimer regenerates the ultraviolet-B photoreceptor UV RESISTANCE LOCUS8 in intact Arabidopsis plants. Plant Physiol.

[43] Sun B, Zhu Z, Cao P, Chen H, Chen C, Zhou X, Mao Y, Lei J, Jiang Y, Meng W (2016). Purple foliage coloration in tea (*Camellia sinensis* L.) arises from activation of the R2R3-MYB transcription factor CsAN1. Sci Rep-UK.

[44] Wu Q, Chen Z, Sun W, Deng T, Chen M (2016). De novo sequencing of the leaf transcriptome reveals complex light-responsive regulatory networks in *Camellia sinensis* cv. *Baijiguan*. Front Plant Sci.

[45] Zhao X, Wang P, Li M, Wang Y, Jiang X, Cui L, Qian Y, Zhuang J, Gao L, Xia T (2017). Functional characterization of a new tea (*Camellia sinensis*) flavonoid glycosyltransferase. J Agr Food Chem..

[46] Cui L, Yao S, Dai X, Yin Q, Liu Y, Jiang X, Wu Y, Qian Y, Pang Y, Gao L (2016). Identification of UDP-glycosyltransferases involved in the biosynthesis of astringent taste compounds in tea (*Camellia sinensis*). J Exp Bot.

[47] Dai X, Zhuang J, Wu Y, Wang P, Zhao G, Liu Y, Jiang X, Gao L, Xia T (2017). Identification of a flavonoid glucosyltransferase involved in 7-OH site glycosylation in tea plants (*Camellia sinensis*). Sci Rep.

[48] He X, Zhao X, Gao L, Shi X, Dai X, Liu Y (2018). Isolation and characterization of key genes that promote flavonoid accumulation in purple-leaf tea (*Camellia sinensis* L.). Sci Rep.

[49] Wei C, Yang H, Wang S, Zhao J, Liu C, Gao L, Xia E, Lu Y, Tai Y (2018). Draft genome sequence of *Camellia sinensis var. sinensis* provides insights into the evolution of the tea genome and tea quality. P Natl Acad Sci USA.

[50] Liu L, Gregan S, Winefield C, Jordan B (2015). From UVR8 to flavonol synthase: UV-B-induced gene expression in sauvignon blanc grape berry. Plant Cell Environ.

[51] Liu L, Gregan SM, Winefield C, Jordan B (2018). Comparisons of controlled environment and vineyard experiments in sauvignon blanc grapes reveal similar UV-B signal transduction pathways for flavonol biosynthesis. Plant Sci.

[52] Chagné D, Allan AC (2013). An ancient duplication of apple MYB transcription factors is responsible for novel red fruit-flesh phenotypes. Plant Physiol.

[53] Jordan B, Jordan B (2017). The effects of ultraviolent-B on *Vitis vinifera*–how important is UV-B for grape biochemical composition?. UV-B radiation and plant life: molecular biology to ecology.

[54] Henry-Kirk Rebecca A., Plunkett Blue, Hall Miriam, McGhie Tony, Allan Andrew C., Wargent Jason J., Espley Richard V. (2018). Solar UV light regulates flavonoid metabolism in apple (Malus x domestica). Plant, Cell & Environment.

[55] Wang W, Zhou Y, Wu Y, Dai X, Liu Y, Qian Y, Li M, Jiang X, Wang Y, Gao L, Xia T (2018). Insight into catechins metabolic pathways of *Camellia sinensis* based on genome and transcriptome analysis. J Agric Food Chem.

[56] Hichri I, Barrieu F, Bogs J, Kappel C, Delrot S, Lauvergeat V (2011). Recent advances in the transcriptional regulation of the flavonoid biosynthetic pathway. J Exp Bot.

[57] Xu W, Dubos C, Lepiniec L (2015). Transcriptional control of flavonoid biosynthesis by MYB-bHLH-WDR complexes. Trends Plant Sci.

[58] Xia E, Zhang H, Sheng J, Li K, Zhang Q, Kim C, Zhang Y, Liu Y, Zhu T, Li W (2017). The tea tree genome provides insights into tea flavor and independent evolution of caffeine biosynthesis. Mol Plant.

[59] Czemmel S, Stracke R, Weisshaar B, Cordon N, Harris NN, Walker AR, Robinson SP, Bogs J (2009). The grapevine R2R3-MYB transcription factor VvMYBF1 regulates flavonol synthesis in developing grape berries. Plant Physiol.

[60] Shi CY, Yang H, Wei CL, Yu O, Zhang ZZ, Jiang CJ, Sun J, Li YY, Chen Q, Xia T (2011). Deep sequencing of the *Camellia sinensis* transcriptome revealed candidate genes for major metabolic pathways of tea-specific compounds. BMC Genomics.

[61] Li CF, Zhu Y, Yu Y, Zhao QY, Wang SJ, Wang XC, Yao MZ, Luo D, Li X, Chen L (2015). Global transcriptome and gene regulation network for secondary metabolite biosynthesis of tea plant (*Camellia sinensis*). BMC Genomics.

[62] Tai Y, Wei C, Yang H, Zhang L, Chen Q, Deng W, Wei S, Zhang J, Fang C, Ho C (2015). Transcriptomic and phytochemical analysis of the biosynthesis of characteristic constituents in tea (*Camellia sinensis*) compared with oil tea (*Camellia oleifera*). BMC Plant Biol.

[63] Melanie B, László K-B, Kata T, Lieven De V, Ferenc N, Roman U (2014). UV-B-responsive association of the Arabidopsis bZIP transcription factor ELONGATED HYPOCOTYL5 with target genes, including its own promoter. Plant Cell.

[64] Christie JM, Arvai AS, Baxter KJ, Heilmann M, Pratt AJ, O’Hara A, Kelly SM, Hothorn M, Smith BO, Hitomi K (2012). Plant UVR8 photoreceptor senses UV-B by tryptophan-mediated disruption of cross-dimer salt bridges. Science.

[65] Wu D, Hu Q, Yan Z, Chen W, Yan C, Huang X, Zhang J, Yang P, Deng H, Wang J (2012). Structural basis of ultraviolet-B perception by UVR8. Nature.

[66] Cloix C, Kaiserli E, Heilmann M, Baxter KJ, Brown BA, O'Hara A, Smith BO, Christie JM, Jenkins GI (2012). C-terminal region of the UV-B photoreceptor UVR8 initiates signaling through interaction with the COP1 protein. P Natl Acad Sci USA.

[67] Konishi S, Miyamoto S, Taki T (1985). Stimulatory effects of aluminum on tea plants grown under low and high phosphorus supply. Soil Sci Plant Nutr.

[68] Grabherr MG, Haas BJ, Yassour M, Levin JZ, Thompson DA, Amit I, Xian A, Fan L, Raychowdhury R, Zeng Q (2011). Trinity: reconstructing a full-length transcriptome without a genome from RNA-Seq data. Nat Biotechnol.

[69] Wang W, Xin H, Wang M, Ma Q, Wang L, Kaleri NA, Wang Y, Li X (2016). Transcriptomic analysis reveals the molecular mechanisms of drought-stress-induced decreases in *Camellia sinensis* leaf quality. Front Plant Sci.

